# A picture is worth a thousand diffraction spots: using photometry and computer vision to perform rapid high-throughput sample-versatile serial crystallography

**DOI:** 10.1107/S2052252526003568

**Published:** 2026-05-18

**Authors:** Ben A. Coulson, Sam G. Lewis, Christian Orr, Lauren E. Hatcher, David R. Allan, Mark Warren

**Affiliations:** a Diamond Light Source Harwell Science and Innovation Campus Didcot Oxfordshire OX11 0DE United Kingdom; b School of Chemistry Cardiff University Main Building Park Place CF10 3AT United Kingdom; Australian Nuclear Science and Technology Organisation and University of Wollongong, Australia

**Keywords:** serial crystallography, photometry, computer vision, high throughput

## Abstract

The ‘photometric selection’ approach as a high throughput, sample tolerant, low cost and highly automatable method of carrying out serial crystallography is presented. Crystalline samples are loaded and distributed onto a simple transparent substrate and an in-line camera identifies crystals using image recognition algorithms from the computer vision project OpenCV. In contrast to established serial techniques, which generally require that crystal samples be refined with narrow size distributions and defined habits, the sample requirements when using photometric selection are shown to be minimal. We demonstrate how broadly effective photometric selection can be by collecting high-quality datasets from three exemplar systems: a small-mol­ecule organometallic, a small-mol­ecule organic and a metal–organic framework system. In contrast to previously established grid-scanning techniques, data collection using photometric selection can be up to six times faster.

## Abstract 2

Computer vision algorithms are employed *in situ* to locate crystal targets for small-mol­ecule fixed-target crystallography. This methodology is shown to be significantly faster and of comparable data quality to previously published methods.

## 1. Introduction

The availability of ultrabright X-ray sources for structure determination is increasing (Chapman, 2023 ▸; Raimondi *et al.*, 2023 ▸). This increased brilliance allows X-ray crystallography studies of increasingly small or weakly diffracting crystals to be carried out and, as a result, experiments studying ultrafast processes with high quantities of dose delivery to samples are becoming more accessible. However, with higher X-ray brilliance comes an increased chance of radiation damage during data collection and individual crystals may not survive for long enough in the beam to provide sufficient data for a full structure elucidation (Lewis *et al.*, 2025 ▸).

Serial crystallography is widely used by the macromolecular crystallography community to probe single diffraction images from a large number of crystals. Indexed data from these crystals is then merged to obtain a complete crystal structure, comparable to a single-crystal rotation dataset (Chapman *et al.*, 2011 ▸). Samples can be mounted onto fixed-target grids, rotating cassette tapes, within sheet-on-sheet chips, polymer meshes or even direct liquid injection into the beam (Mehrabi *et al.*, 2020 ▸; Weierstall, 2014 ▸; Illava *et al.*, 2021 ▸; Zhao *et al.*, 2019 ▸; Cheng, 2020 ▸; Doak *et al.*, 2018 ▸; Cianci *et al.*, 2019 ▸; Doak *et al.*, 2024 ▸). This has several advantages compared to single-crystal studies, including reducing radiation damage by spreading the radiation dose across crystals, with one example claiming the collection of an effectively ‘zero dose’ structure from a radiation-sensitive sample (Pearson & Mehrabi, 2020 ▸; Schulz *et al.*, 2022 ▸; Hough & Owen, 2021 ▸; Ebrahim *et al.*, 2019 ▸; Lewis *et al.*, 2025 ▸).

Several works have recently emerged applying serial techniques to small-mol­ecule crystal systems (Schriber *et al.*, 2022 ▸; Takaba *et al.*, 2023 ▸; Kang *et al.*, 2024 ▸). One key challenge to overcome is the relatively sparse diffraction patterns, making indexing small-mol­ecule systems more difficult compared to macromolecules (Moon *et al.*, 2024 ▸). Introducing a small rotation at each crystal location, through methods such as small rotative fixed-target serial synchrotron crystallography (SR-FT-SSX), tackles this by collecting small wedges of diffraction space for each crystal. This increases the amount of data available, therefore making indexing individual crystal datasets much easier. SR-FT-SSX has been demonstrated as a viable approach to carrying out serial crystallography on small-mol­ecule and framework systems at synchrotrons (Lewis *et al.*, 2024 ▸; De Zitter *et al.*, 2024 ▸).

Generally, serial synchrotron crystallography experiments tend to use grid substrates for sample mounting, which are typically covered with many tapered holes or ‘wells’. These wells are positioned in a precisely ordered array, allowing motors to raster across the surface to probe the contents of each well. Grid substrates have been successfully employed in a large number of serial crystallography studies (Ebrahim *et al.*, 2019 ▸; Mehrabi *et al.*, 2020 ▸; Pearson & Mehrabi, 2020 ▸; Schulz *et al.*, 2022 ▸; Carrillo *et al.*, 2023 ▸; Roedig *et al.*, 2016 ▸; Roedig *et al.*, 2017 ▸; Owen *et al.*, 2023 ▸; Ren *et al.*, 2018 ▸; Lee *et al.*, 2020 ▸; Horrell *et al.*, 2021 ▸; Sherrell *et al.*, 2022 ▸). In this work, we demonstrate the serial crystallography of samples mounted onto planar ‘flat-film’ sample holders, with the crystals identified using computer vision and photometric selection for the first time.

Sample loading onto a planar film provides a more facile alternative to grid loading with several advantages. Firstly, it allows SR-FT-SSX to be applied to samples without going through lengthy and sample-costly recrystallization studies. In fact, in this work, we demonstrate that it is possible to use relatively heterogenous samples, including those ‘as obtained’ from chemical suppliers. Secondly, mounting the crystals directly onto a flat film also means that the samples on the substrate are more representative of the bulk, with none of the size or shape filtering that occurs during loading or recrystallization. Thirdly, in comparison to textured grid substrates, flat-film sample holders are cheaper and quicker to produce and load at scale, whilst being compatible with robotic manipulators and liquid-nitro­gen-flow cooling devices with no further modification.

As a result of these three advantages, we tentatively suggest that our methodology will enable the study of small-mol­ecule systems that previously were considered unsuitable for serial experiments. It is our hope that by demonstrating the range of samples SR-FT-SSX can successfully study in this work, we will encourage a wider uptake of the technique in the small-mol­ecule community.

## 2. Methods

### 2.1. Sample mounting

**Figure 1:**
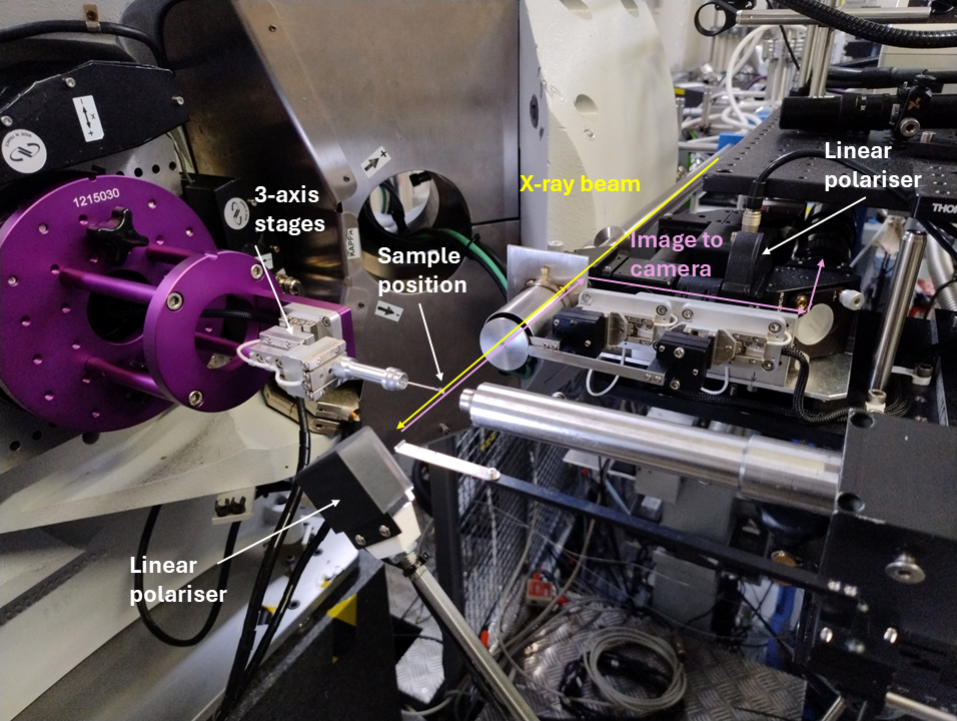
Photograph of the experimental setup at I19 at the UK Synchrotron Diamond Light Source, showing the sample in the X-ray path, the camera setup to take in-line photographs of the sample and the position of the polarizers for capturing birefringence.

All data were collected at I19 at the UK Synchrotron Diamond Light Source on a four-circle Newport Dif­frac­tom­eter in EH2, equipped with an Eiger 4M CdTe photon-counting pixel array detector and with an X-ray wavelength of 0.4859 Å (Ag K-edge). The sample was mounted normal to the X-ray beam on top of three linear piezo stages from SmarAct SLC2430, allowing for translating orthogonal to the X-ray beam path and translation along the X-ray beam path (Fig. 1 ▸). Temperature control was carried out using an Oxford Cryostream 700 series.

Custom flat-film sample holders were laser cut from a 50 µm-thick poly(methyl methacrylate) (PMMA) film using a custom in-house laser shaping system and were glued to standard single-crystal pin magnetic bases. The sample area is 2.88 mm × 2.88 mm with 50 µm diameter fiducial holes at each corner (Fig. S2 in the supporting information). These flat-film sample holders are low cost and are compatible with both standard liquid-nitro­gen-flow cooling devices and sample robot systems, which will allow for the planned future automation of the process. Crystalline samples are dispersed in manipulation oil (Fomblin Y) and spread evenly across the PMMA sample holders using a Mylar ‘rake’ tool (Fig. S3) to allow good dispersal of the crystals without damaging the sample. The flat film is mounted on the diffractometer (Fig. 1 ▸) and motorized stages are used to align the sample film using three fiducial points, *i.e.* in the top-left, top-right and bottom-left corners. By using three orthogonal motor stages, the fiducials are each brought to the centre of rotation about phi, which is in turn aligned with the X-ray beam. The motor positions that place each fiducial into the centre of rotation are then used to generate a set of motor positions across the sample that are also aligned with the centre of rotation. This allows large rotations about phi to be carried out at any point on the sample without significantly moving the crystal relative to the X-ray beam. All data were collected with a 100 µm beam; however, it should be noted that the beamline at I19 is equipped with multiple pinhole sizes (with diameters of 20, 40 and 100 µm; see examples in Fig. S4), allowing the beam size and flux level to be tuned to the sample where necessary.

### 2.2. Sample preparation

[1,2-Bis(di­phenyl­phosphino)ethane]­dichloro­nickel (**1**) was synthesized according to the literature (Warren *et al.*, 2014 ▸). 4′-Chloro-2,2′:6′,2′′-terpyridine (**2**) was purchased from Sigma–Aldrich and used as received. Tri­aqua­bis­(benzene-1,3,5-tri­car­boxyl­ato)tricopper(II) (**3**) was prepared by Conor Rowley using a published method while working with Professor Cameron Kepert at the University of Sydney (Wu *et al.*, 2010 ▸).

### 2.3. *In situ* crystal finding

**Figure 2:**
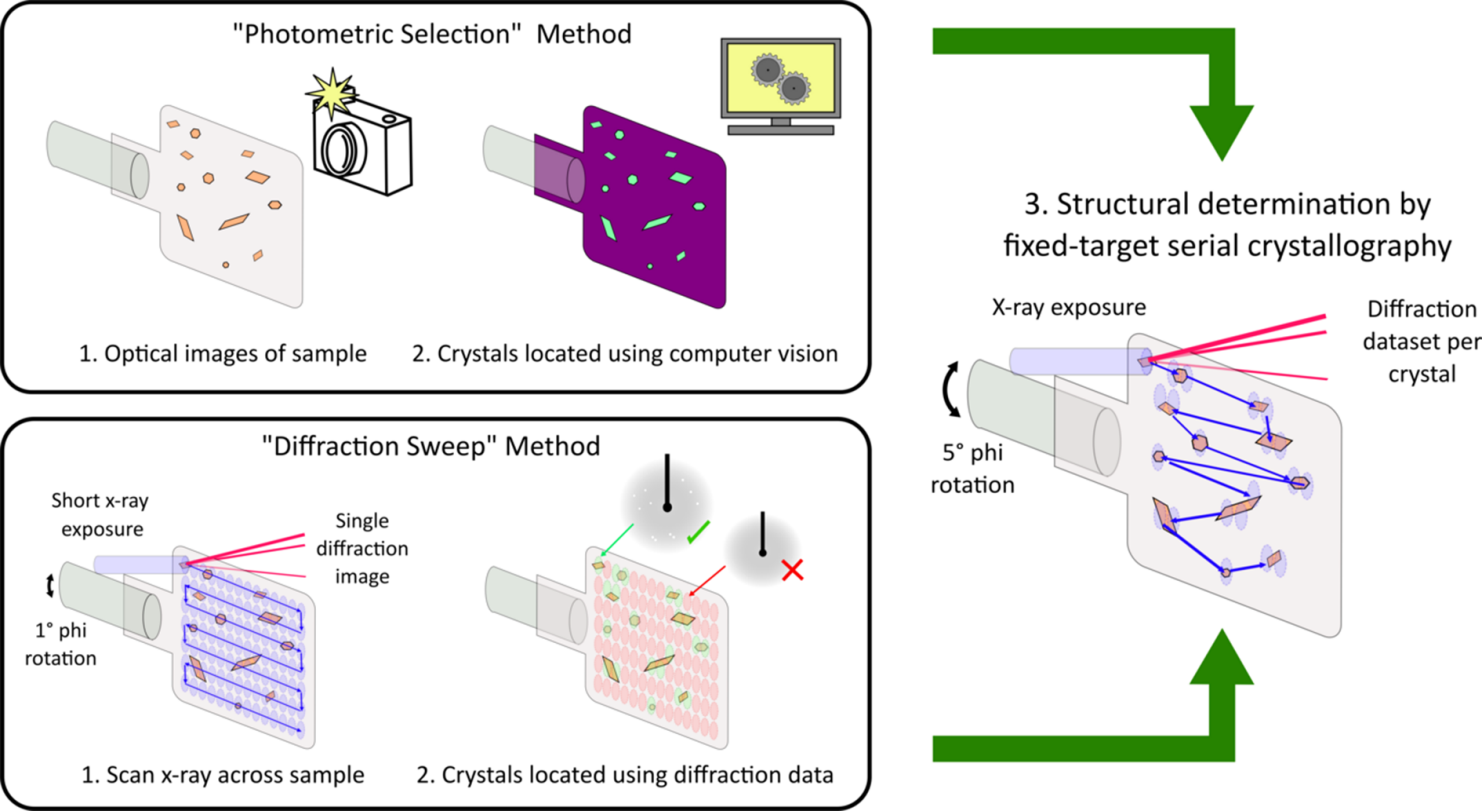
An overview of how the steps were taken, showing how the photometric selection (PS) method compared to the diffractive sweep (DS) method in finding crystals on a flat planar substrate.

The principal challenge presented when a sample is mounted onto a planar film lies in locating the positions of individual crystals. In contrast to grid-based mounts or liquid-injection systems, where the jet or the well parameters pre­define a finite number of positions where crystals may be found, the dispersion of crystals across a flat film is more ran­dom. We present a method, hereafter known as the ‘Photometric Selection’ (PS) method, which involves taking an optical still image of the sample and applying image-processing and computer vision techniques to detect the positions of the crystals (Fig. 2 ▸). In contrast to previously published fixed-target techniques, this technique requires neither an initial X-ray exposure nor a lengthy scan, thus speeding up the overall collection process and minimizing the total X-ray dose experienced per crystal. To assist in identifying crystals, our in-line microscope camera system has been adapted to include two linear polarizers, allowing us to view any birefringence the sample exhibits *in situ* (Fig. 1 ▸ and Fig. S1 of the supporting information).

Evaluating crystals photometrically is not always intuitive and so we compare PS to a more conventional ‘grid-scanning’ serial technique to ensure that the advantages of locating crystals with PS do not come with a significant loss in data quality (Wojdyla *et al.*, 2016 ▸; De Zitter *et al.*, 2024 ▸). We do this using a technique we refer to as the ‘Diffractive Sweep’ technique (DS), *vide infra*.

### 2.4. Photometric data collection

**Figure 3:**
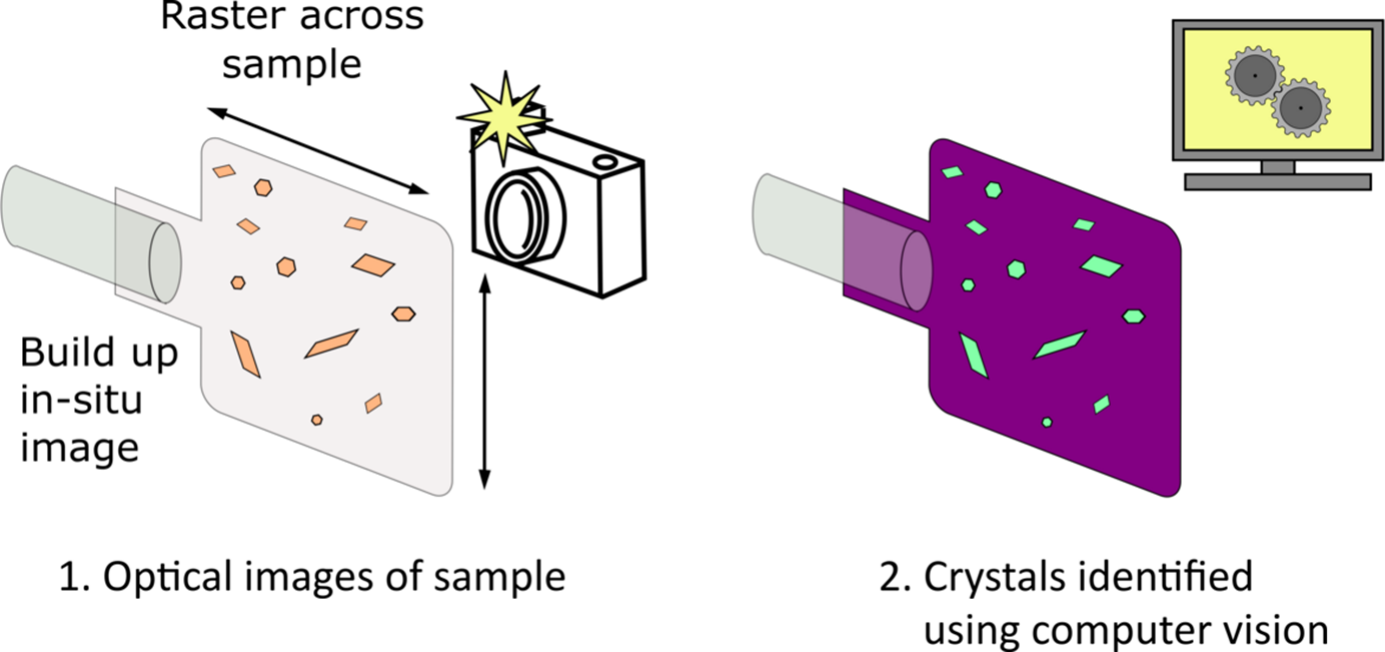
The locations of crystals on a planar substrate are found by taking optical images of the entire substrate and then using computer vision algorithms to identify the crystals in the image.

To identify crystals using photometric selection (PS), after fiducial alignment, an on-axis camera (a Mako G-234 C in a bespoke lens assembly) was used to record a series of optical still images of the substrate which was mounted on the goniometer head. The substrate was then rastered in front of the camera and a 6×8 grid of photographs taken until the entire substrate had been imaged (Fig. 3 ▸). By storing the motor movements between the fiducials and relating them to known scaling factors, a composite image was generated by collecting the still images together. Alongside this composite image, a human-readable file was generated relating every pixel in the composite image to a set of real-space goniometer motor positions.

### 2.5. Image processing with OpenCV

**Figure 4:**
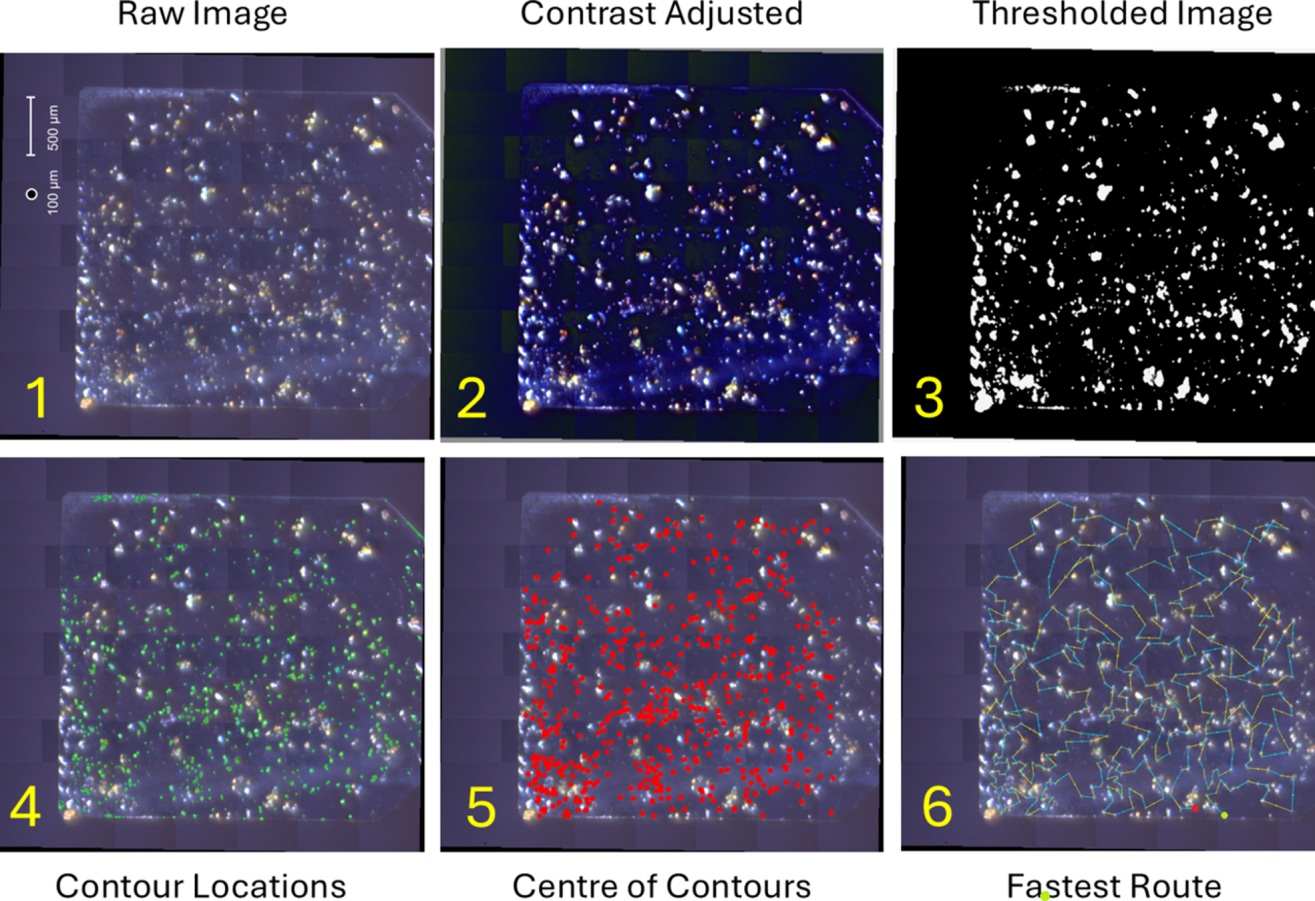
Image-processing pipeline for photometric crystal identification on a flat film. (1) From the original image, with a 500 µm scale bar and key to indicate a 100 µm beamsize relative to the sample; (2) this was processed in a multistep procedure comprised of adjusting the contrast of the image; (3) thresholding the image to generate a binary image setting all the pixel values above a certain value at 1 and all other values as 0; (4) using OpenCV’s ‘findContours’ algorithm to locate the centre of crystals; (5) superimposing the positions of the crystals located in the previous step onto the raw image; and (6) finding the shortest pathway across the sample which visits each crystal location once. For expanded (6), see Fig. S7 in the supporting information.

Composite images are analysed using a bespoke piece of software written in Python. A graphical user interface was developed in-house using the PyQt library and is intended for non-expert beamline users. The image-processing software also utilized OpenCV, an open-source computer vision package (Bradski, 2000 ▸; The OpenCV Library, https://opencv.org/). The general steps for using the software are outlined in Fig. 4 ▸. The open-source software used, along with example images, are available according to the data availability statement. Full details of the parameters used for the samples shown in this work can be found in Figs. S6, S12 and S18, and corresponding Tables S1, S5 and S9 of the supporting information.

Note that the example substrate is held between two crossed linear polarizers and, for some of the samples in this work, certain crystals appear as bright regions due to their birefringence. Starting from the raw composite image (Fig. 4 ▸, Section 1), the contrast and brightness are initially adjusted to make those bright regions stand out as much as possible from the background (Fig. 4 ▸, Section 2). This adjusted image is then converted into a binary image, with pixels above a variable threshold being converted into a ‘white’ pixel and those below that threshold converted to a ‘black’ pixel (Fig. 4 ▸, Section 3). In this step, a small amount of Gaussian blurring is carried out to smooth the edges of the binarized regions.

This binary image is then fed into the ‘findContours’ function in the OpenCV library, which locates isolated regions of the same colour within the binary image (The OpenCV Library, https://opencv.org/; Bradski, 2000 ▸). These contours are overlaid onto the raw image (Fig. 4 ▸, Section 4) to allow the user to see which regions are identified as bright (*i.e.* assumed to be crystalline). At this stage, we apply size-based filtering to remove any artefacts, multiple crystals, anomalous photographic artefacts or significantly oversized crystals. Following this size filtering, the centres of the contours are recorded as pixel values and overlaid onto the raw image as a small red point (Fig. 4 ▸, Section 5).

Finally, a function designed to solve the Travelling Salesman problem (from Python TSP Solver) is applied to the pixel coordinates to reduce the path length around the substrate compared to a ‘rasterized’ route (Fig. 4 ▸, Section 6) (Goulart, 2024 ▸). In the case shown in this example, the final route is around 11% of the distance of a raster route, so overheads are reduced significantly by this final step. It should be noted here that a heuristic function is applied rather than one intended to find a true solution. This significantly reduces the computational cost; however, this also means that a different ‘optimal’ path is produced each time the data are processed (Goulart, 2024 ▸).

The final output is a list of image pixel positions, ordered to minimize travel time between them. As stated previously, each pixel position is linked to specific motor positions, and so it is trivial to convert these pixel positions into motor positions to move the crystals into the beam for experimental collection.

### 2.6. Diffractive sweep crystal identification

**Figure 5:**
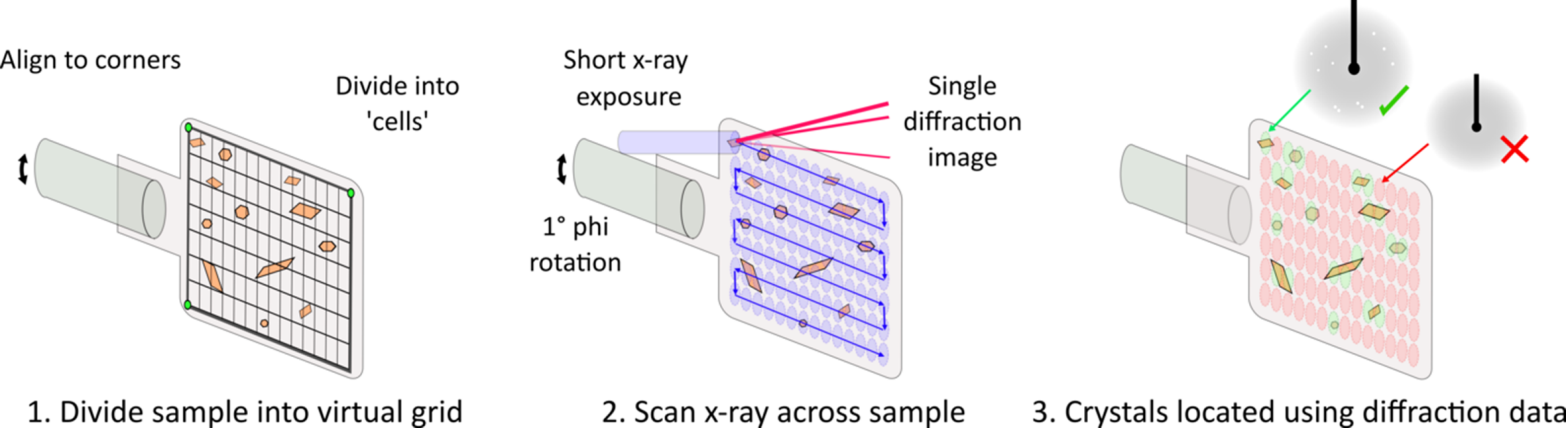
Identifying crystal positions on a flat substrate using the diffractive sweep method involves dividing the substrate into *n* × *n* (*n* = 36) virtual cells, then carrying out an initial ‘spot-finding’ coarse data collection with a single image per cell. The number of diffraction spots per cell are counted and used to identify the cells containing crystals.

To identify crystals using the diffractive sweep method, after fiducial alignment, the sample is divided into a grid of *n* × *n* virtual cells (Fig. 5 ▸). For the samples investigated in this work, *n* = 36 (*i.e.* a total of 1296 cells); however, this value can be tuned according to experimental demands, with a smaller beam size requiring a higher number of cells to maintain full sample coverage. For example, using the 40 µm pinhole increases the number of cells to 9216. The sample is then rastered across the X-ray beam such that each cell is subject to a single 0.5 s X-ray image collection taken across a 1° phi rotation. The software package *Diffraction Integration for Advanced Light Sources* (*DIALS*) is then used to count the number of diffraction spots observed per cell (Winter *et al.*, 2018 ▸). The coarse images from these ‘spot-finding’ experiments are used to determine the presence of any diffracting material on the substrate and no further peak processing is carried out at this stage.

**Figure 6:**
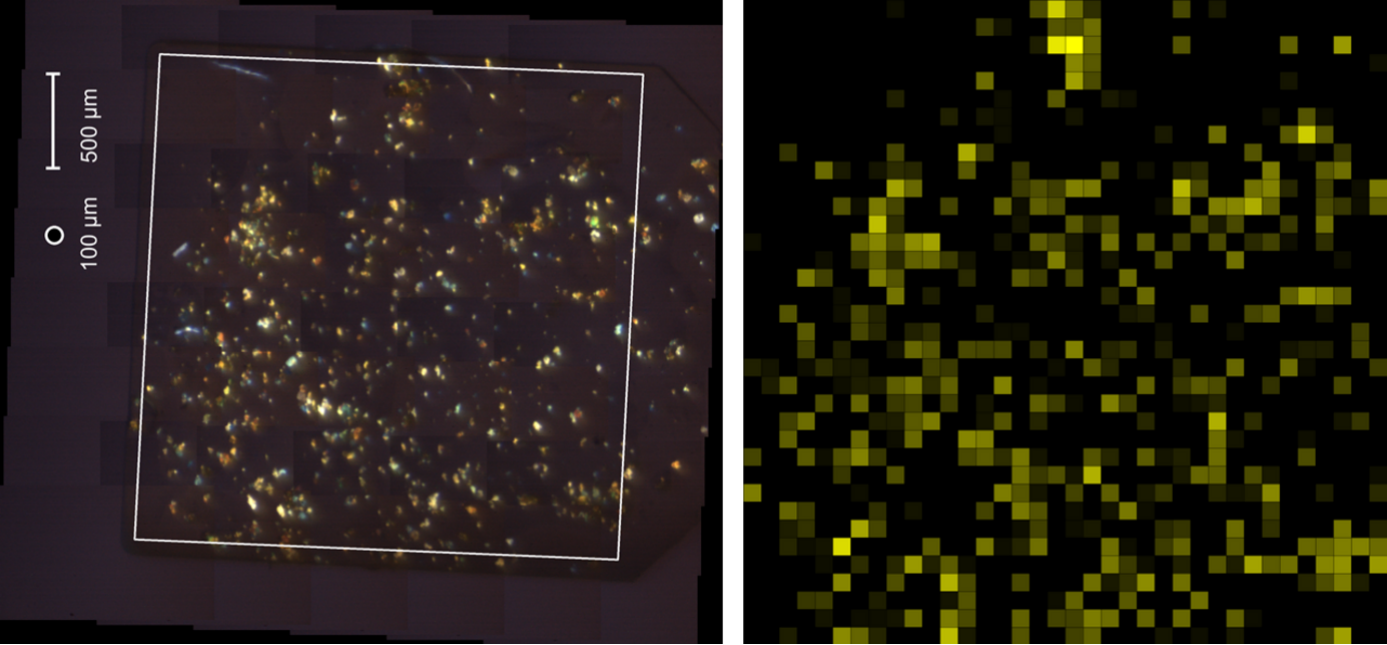
Microscope images of the substrate loaded with **1** (left), compared to the ‘diffraction map’ (right) reconstructed from a ‘spot-finding’ collection, where single diffraction images are taken at each pixel. Pixel brightness (in yellow) corresponds to the number of diffraction spots at that cell, whereas black areas indicate no diffraction spots were recorded. The area on the photograph corresponding to the collection area is denoted by the white box.

Cells corresponding to images containing a total number of spots between selected thresholds (in general, any number above 10 and lower than 100) are then selected for further data collection. This data can also be used to generate spatially resolved ‘diffraction maps’, where an image of the sample is built up by converting the number of spots observed for each well into a ‘pixel brightness’. These diffraction maps show good agreement with microscope images of the samples (Fig. 6 ▸ and Figs. S8, S14 and S20 of the supporting information).

### 2.7. Processing diffraction data

Each individual crystal located according to either DS or PS is subjected to a 5° partial rotation dataset collection, with 25 images taken at 0.2° per image and 0.2 s of exposure per image. Each dataset is processed separately using an auto-processing pipeline for SR-FT-SSX data, outlined in a previous publication (Lewis *et al.*, 2024 ▸). The pipeline utilizes *DIALS* implemented through *Xia2* and is semi-automated with in-house scripts (Winter *et al.*, 2018 ▸). Spot-finding and indexing is carried out to determine the unit-cell parameters and space group.

Following our previous work, this processing occurs according to a simple feedback loop (Lewis *et al.*, 2024 ▸). First, an initial sweep of the data allowing *DIALS* to determine the unit cell by itself is usually sufficient to learn the unit-cell parameters of an unknown sample. The processing is then repeated, feeding the known unit-cell parameters and space group into *Xia2*. In this way, a higher number of rotation datasets are successfully solved in the second pass. Occasionally, multiple lattices are observed within the same collection, which is a symptom of the relatively large beam size compared to the sample (Fig. 6 ▸). In this case, the largest component is extracted from these collections and the remaining data are discarded. Previous works have demonstrated that a multi-lattice indexer can extract more data; however, high-quality data were obtained without the need for this technique (Beilsten-Edmands *et al.*, 2024 ▸).

A selection of refinement metrics from each solved dataset (including indexed unit-cell parameters, space group, diffraction signal-to-noise and residual *R* factors) is extracted from the individual datasets and used as ‘structure quality factors’ to assess the quality of each partial dataset. The datasets can then be filtered according to chosen metrics to ensure the highest quality data are taken forward for merging and scaling into the final dataset. In this work, *I*/σ(*I*) and *R*_pim_ were used as the primary quality criteria (see Section S1 in the supporting information). Individual rotation datasets meeting the quality criteria were then merged using *DIALS* before a final cell refinement and structure solution and refinement using *SHELXT* and *SHELXL*, respectively (Winter *et al.*, 2018 ▸; Sheldrick, 2015*a* ▸; Sheldrick, 2015*b* ▸). Note that for serial crystallography datasets, it is often preferable to use *R*_pim_ rather than *R*_int_. Variations in crystal size and partiality affect individual measurements within a serial crystallography dataset, but averaging many observations improves the overall precision. Therefore, in serial crystallography it is desirable to collect large amounts of data and the datasets often exhibit high multiplicity. However, *R*_int_ increases with multiplicity even when data quality is good, making *R*_int_ a misleading data quality metric in this context. *R*_pim_ is a metric related to *R*_int_, which also includes a multiplicity correction and therefore provides a fairer and more meaningful measure of data quality when referring to serial crystallography datasets (Weiss, 2001 ▸). It is critical to bear in mind that *R*_pim_ and *R*_int_ are not the same metric and cannot be directly compared – for example, an *R*_pim_ of 0.032 does not indicate the same data quality as would an *R*_int_ of 0.032. For the datasets presented in this work, the *R*_pim_ observed is within acceptable parameters, indicating good overall data quality despite the high *R*_int_.

## 3. Results

### 3.1. [1,2-Bis(diphenyl­phosphino)ethane]­dichloro­nickel (1)

Sample **1** was selected as an organometallic system for analysis with this technique. It contains a first-row transition metal and is established in the literature for its catalytic properties (Clevenger *et al.*, 2020 ▸). Additionally, **1** crystallizes in the space group *P*2_1_/*c* [which accounts for 33.8% of structures in the Cambridge Structural Database (CSD; Groom *et al.*, 2016 ▸) as of April 2025 ▸] and the relatively low symmetry of the system can provide insights as to how best to collect serial datasets to result in a high-completeness structure. For these two reasons, **1** can be considered a representative exemplar of organometallic samples.

**Figure 7:**
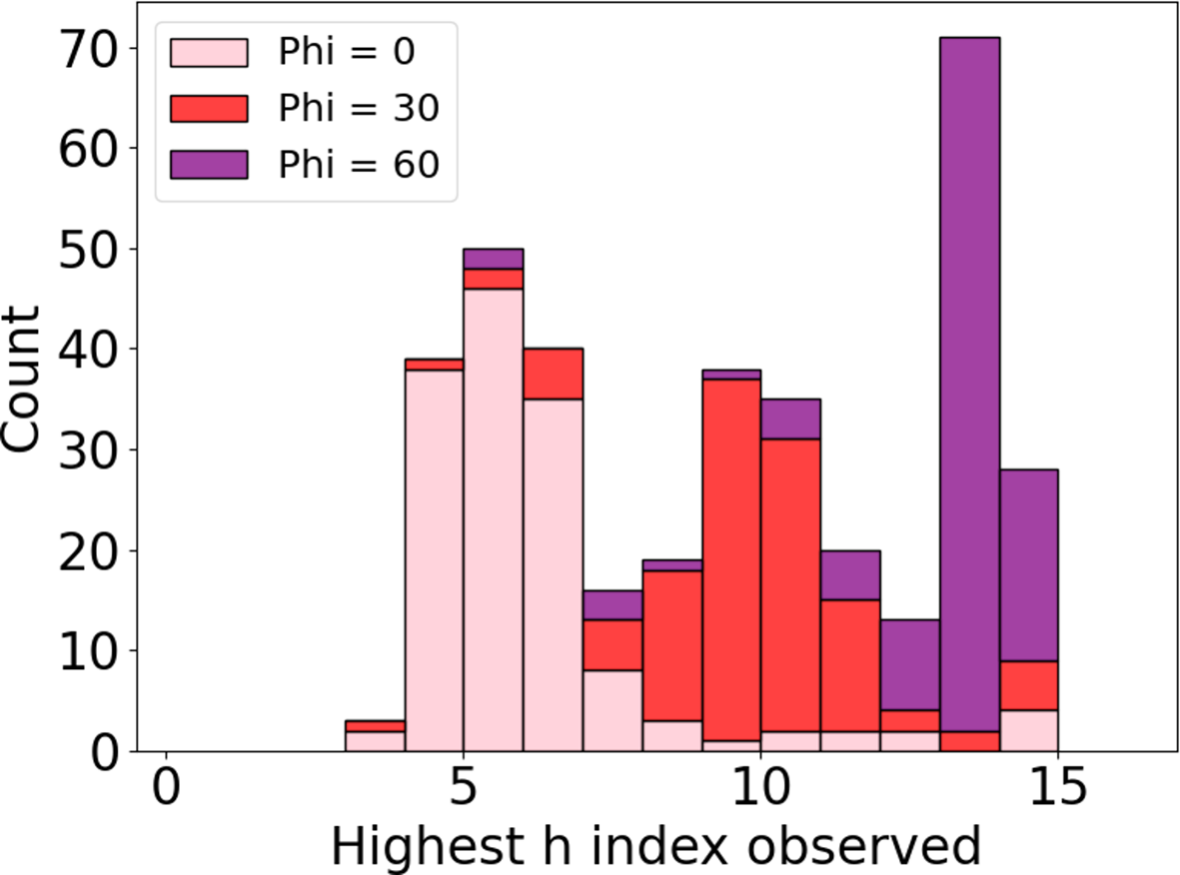
A representation of how collections at different phi angles can increase the data coverage obtained from crystals loaded onto a planar film in serial crystallography experiments.

Initially, the 5° rotation datasets were collected around a centre phi position of 0° (*i.e.* with the normal of the substrate parallel to the incident X-rays); however, the resulting merged datasets were of low completeness. This results from crystals of **1** having a preferred orientation on the planar substrate, which only enabled the collection of reflections with lower *h* indices close to phi = 0° (Fig. 7 ▸ and Fig. S5 of the supporting information). In previous and other published works, substrates contain wells, small cavities in which crystals are likely to adopt a wider and more random variety of orientations, and so a higher completeness is obtained.

**Figure 8:**
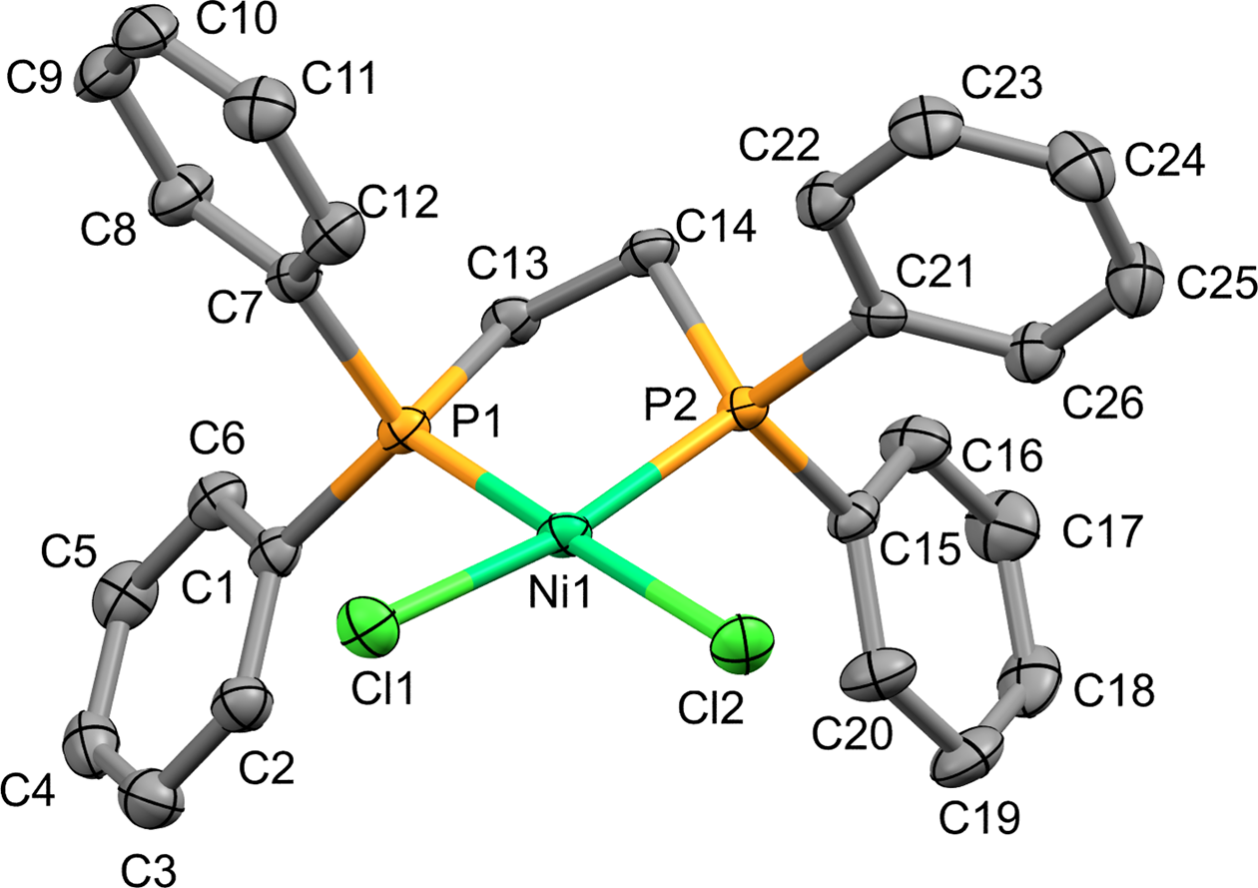
The crystal structure of **1**, with the crystal located by the PS method. Displacement ellipsoids are drawn at the 50% probability level and H atoms have been omitted for clarity. Additional data are outlined in Table 1 ▸.

Rather than complicate our simple flat substrate design, we chose to simulate the random orientation of crystals within wells by varying the initial phi position of the entire substrate. As the angle between the incident beam and the normal to the substrate is increased, we see that higher *h* indices are accessible across the 5° scan (Fig. 7 ▸). It was found that a good coverage of *h* indices was obtained by combining the data from 5° rotation datasets collected around phi angles of 0, 30 and 60°, with each dataset coming from a different crystal (Fig. 7 ▸). The merged dataset is of good quality and high completeness (Fig. 8 ▸ and Table 1 ▸). Therefore, with no additional equipment, processing costs or time investment, we built up a high-completeness dataset in the presence of strong preferred orientation effects. This multi-phi position approach is widely applicable to other fixed-target collections where completeness is low.

The same substrate and sample were then used to perform a comparable DS SR-FT-SSX data collection. Table 1 ▸ allows direct comparison of the data obtainable by DS and PS. Both datasets are of high quality with good *I*/σ(*I*) and residual factors, although the data from the DS method tends to be slightly better in comparison to the PS method. Note that for serial crystallography datasets, it is preferable to compare *R*_pim_ rather than the usual *R*_int_ often used as a metric for single-crystal data (see supporting information Section S1). Of note is that the fraction of datasets collected that were used in the final merged structure [*i.e.* those with a suitable *I*/σ(*I*) and *R*_pim_] is higher for DS (42%) compared to PS (28%). This is likely a result of DS finding crystals by directly selecting according to diffraction behaviour. However, even accounting for the slightly higher amount of data required, the fact that no preliminary scan is required still means that the overall time taken to collect data using the PS method is shorter than data collection with a comparable DS method. The data quality of both multi-crystal structures are comparable to a previously published single-crystal structure (Busby *et al.*, 1993 ▸).

### 3.2. 4′-Chloro-2,2′:6,2′′-terpyridine (2)

Following the successful data obtained for organometallic compound **1**, an exemplar organic compound was chosen as the next candidate for study. **2** was selected as it is a commercially available organic compound which is of inter­est as a ligand for developing catalysts and other novel inorganic compounds (Karges *et al.*, 2019 ▸). Conveniently, **2** can be obtained commercially in a crystalline form and was used as received, with no additional recrystallization stages required prior to data collection.

The data obtained from crystals of **2** were of slightly lower quality than those collected from **1**; however, the data quality was generally still good, with high completeness and low residual factors com­parable to that of a previously published single-crystal structure (Beves *et al.*, 2006 ▸). Inter­estingly, the fraction of the collected datasets that was used to provide the final merged structure using the DS technique for **2** is the same as that used for **1** at *ca* 40%. However, compared to **1**, the overall number of datasets collected (and those that passed the filtering criteria) is lower. Even when considering that **2** crystallizes in the orthorhom­bic space group *Pna*2_1_, the completeness for **2** is slightly lower than for **1** due to the lower number of suitable datasets. However, the data for **2** still fall within acceptable parameters, even after the *I*/σ(*I*) filter limit is tuned down from 3 to 2 for the PS dataset (Table 2 ▸).

### 3.3. Tri­aqua­bis­(benzene-1,3,5-tri­carboxyl­ato)tricopper(II) (3)

Framework materials, especially metal–organic frameworks (MOFs), have exploded in popularity in recent years, with the 2025 ▸ Nobel Prize in Chemistry recently awarded for their discovery and development. Our exemplar MOF, sample **3**, is a network of ben­zene­tri­carb­oxy­lic acid ligands and copper metal paddlewheels, also known as HKUST-1, one of the first reported MOFs (Chui *et al.*, 1999 ▸). Crystallography experiments on MOFs are complicated by their porous structure and can require that crystals are mounted in mother liquor or specific solvents to prevent pore collapse. In principle, photometric selection techniques can be applied to such systems with minimal complications, as the substrate has been designed to be compatible with liquid-nitro­gen-flow devices for facile temperature control across the entire sample.

**3** crystallizes in the cubic space group *Fm*
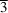
*m*. Due to the high symmetry of **3**, fewer datasets at fewer starting phi angles are required compared to **1** or **2**. This highlights the importance of the on-the-fly processing of the data to ensure that the correct amount of data is collected for the system under study.

One complication when applying PS techniques to **3** is that cubic systems do not exhibit birefringence, so it may appear that the image-processing pipelines discussed earlier in this work are not able to be used. However, **3** is strongly coloured, and so the crystals can be highlighted in the image by simply inverting the pixel intensities (Table S9 and Fig. S20). Although this will not be a suitable approach for all systems, in the case of **3** an excellent PS dataset was collected. This ‘inverted mode’ of image processing is built into the software used for processing the data and can be applied to any system readily.

Both the PS and DS datasets for **3** (Table 3 ▸) are of good quality with low residuals and good completeness, with data comparable to those of previously published single-crystal structures (Chui *et al.*, 1999 ▸). These structures have been obtained using a solvent mask (BYPASS) to mask away *ca* 27 electrons from the pores – this roughly corresponds to an ethanol mol­ecule, which is highly disordered within the pore structure (van der Sluis & Spek, 1990 ▸).

## 4. Sample independent observations

Having shown that both the PS and the DS methods provide high-quality data, it is worthwhile comparing the techniques more generally across the samples, starting with the time taken for full data collection. A full breakdown of the time required to collect the data for both PS and DS can be found in Tables S14–S16 and Fig. S25; however, in general, data collection is faster in a PS experiment compared to a DS experiment due to the time investment required for the initial grid scan in DS. The difference in the time taken for a PS compared to a DS data collection is most noticeable in samples that do not require many small degree wedges to build up completeness, *i.e.* in **3**, with space group *Fm*
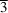
*m*, where a PS collection is over six times faster than the corresponding DS. Even for the relatively low-symmetry samples **1** and **2**, the time savings are significant. Another consideration is that the time taken for a DS data collection depends heavily on the beam size, as the duration of the initial grid scan increases rapidly as you cover the entire substrate, as rather than probing 1296 virtual wells (36 × 36 at 100 µm beamsize), there are instead 9216 virtual wells (96 × 96 at 40 µm beamsize) (Fig. S4 and Tables S17 and S18). In contrast, the time taken to carry out a PS collection has no dependence on the beam size. We anticipate that the time savings of PS compared to DS will continue to improve as we learn more about the scope and limitations of the technique.

One aspect of this work that has not yet been discussed in any significant depth at this point is the size and size distribution of the crystals used. Crystal sizes are calculated from the microphotographs of the substrates (Table 4 ▸ and Fig. S24 and Table S13 of the supporting information), and the data demonstrates that these are crystals on the scale of tens of microns with a wide size distribution. The photometry data indicate that a large number of sub-micron crystals are present, although at this scale no details about the crystal can be made out in the microphotographs. Though small, the average sizes of these samples are larger than our smallest recorded sample to be collected on I19, which was *ca* 1 µm (Aleksich *et al.*, 2025 ▸). Mounting crystals of this size is often the primary challenge in data collection, but using these PMMA mounts, the process is relatively simple and rapid.

In addition to facilitating the mounting of very small crystals, we have also demonstrated that another advantage of PS over DS is that certain crystal sizes can be targeted by applying size filtering in our software. There are several reasons this selectivity is desirable. Ideally, the crystal size should be matched to the beam size. Larger crystals are often more prone to being polycrystalline and suffer from more significant X-ray absorption effects, both of which can reduce the overall quality of the data. It is for these reasons that a conscious effort was made in this work to avoid the largest crystals using the PS method. In contrast to PS, when locating crystals using the DS method, no size filtering can be applied as no size information is gathered. Instead, DS selects crystals based on the number of reflections observed, which is reflected in the higher modal *I*/σ(*I*) and *R*_pim_ for the aggregate datasets of crystals selected by DS compared to PS (Tables S3, S4, S7, S8, S11 and S12). In future applications of this technique, there may also be experiment-specific reasons to target crystals based on size, *e.g.* to target a specific phase or habit of crystals in a mixed-phase sample.

## 5. Conclusion

We have demonstrated that photometric selection of crystals can be carried out *in situ* across a range of small-mol­ecule and framework crystalline samples. Although the planar substrates induce a clear preferred sample orientation, this is overcome by tuning the initial exposure angle to obtain structures with high completeness. Although the data quality is comparable assessing photometric selection to gridscan techniques, there are significant advantages to carrying out PS. Primarily, PS was shown to be up to six times faster than DS methods. Additionally, the PS technique exposes crystals to significantly reduced X-ray flux overall, which is critical when handling radiation-sensitive samples or high X-ray intensity. Therefore, for time-sensitive or radiation-sensitive samples, the practical advantages of PS identification make it a very attractive technique to integrate into workflows. Finally, the sample versatility offered by PS techniques means than many hundreds of crystals can be loaded in seconds and a full bulk analysis of samples can be carried out. We believe that PS has the potential to make serial crystallography more accessible to a wide variety of new audiences and, by developing various automation processes on our beamline, we are opening up the technique to general users with suitable samples.

This work represents a step along the path toward automated tools for SR-SSX, however, there is still room for improvement. Our next steps include the development of more advanced crystal vision techniques and the incorporation of AI and ML to support on-the-fly decision making during data collection.

Our long-term aim is to enable crystals of inter­est to be identified, and diffraction data to be collected and analysed, during the experiment in a fully integrated high-throughput workflow.

## Supplementary Material

Crystal structure: contains datablock(s) global, ni_tsp_3isigma0p2rpim, ni_gridscan_3isigma0p2rpim, cubtc_tsp_isigma3_rpim0p2, cubtc_gridscan_isigma3_rpim0p2, clterp_tsp_isigma2_rpim0p2, clterp_gridscan_isigma3_rpim0p2. DOI: 10.1107/S2052252526003568/oz5012sup1.cif

Structure factors: contains datablock(s) ni_tsp_3isigma0p2rpim. DOI: 10.1107/S2052252526003568/oz5012ni_tsp_3isigma0p2rpimsup2.hkl

Structure factors: contains datablock(s) ni_gridscan_3isigma0p2rpim. DOI: 10.1107/S2052252526003568/oz5012ni_gridscan_3isigma0p2rpimsup3.hkl

Structure factors: contains datablock(s) cubtc_tsp_isigma3_rpim0p2. DOI: 10.1107/S2052252526003568/oz5012cubtc_tsp_isigma3_rpim0p2sup4.hkl

Structure factors: contains datablock(s) cubtc_gridscan_isigma3_rpim0p2. DOI: 10.1107/S2052252526003568/oz5012cubtc_gridscan_isigma3_rpim0p2sup5.hkl

Structure factors: contains datablock(s) clterp_tsp_isigma2_rpim0p2. DOI: 10.1107/S2052252526003568/oz5012clterp_tsp_isigma2_rpim0p2sup6.hkl

Structure factors: contains datablock(s) clterp_gridscan_isigma3_rpim0p2. DOI: 10.1107/S2052252526003568/oz5012clterp_gridscan_isigma3_rpim0p2sup7.hkl

Supporting information. DOI: 10.1107/S2052252526003568/oz5012sup8.pdf

CCDC references: 2501838, 2501835, 2501837, 2501833, 2501834, 2501836

## Figures and Tables

**Figure 9:**
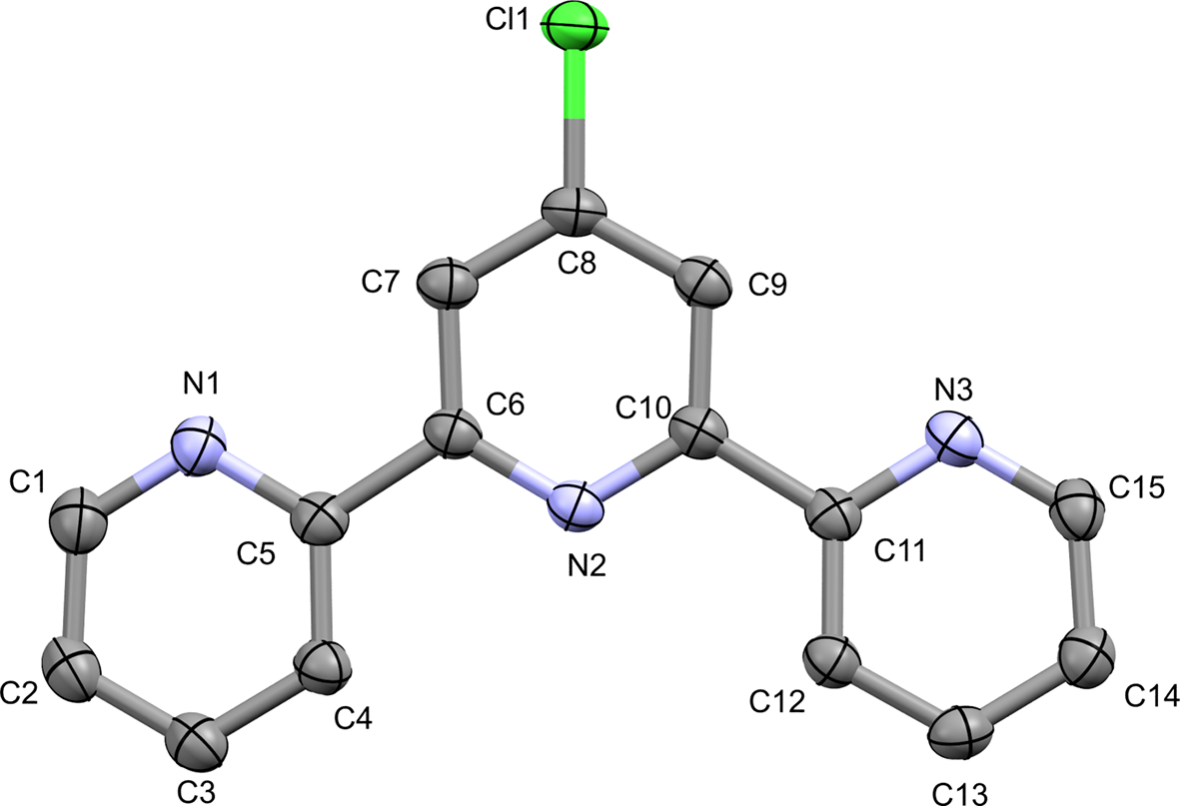
The crystal structure of **2**, with the crystal located by the PS method. Displacement ellipsoids are drawn at the 50% probability level and H atoms have been omitted for clarity. Full data are given in Table 2 ▸.

**Figure 10:**
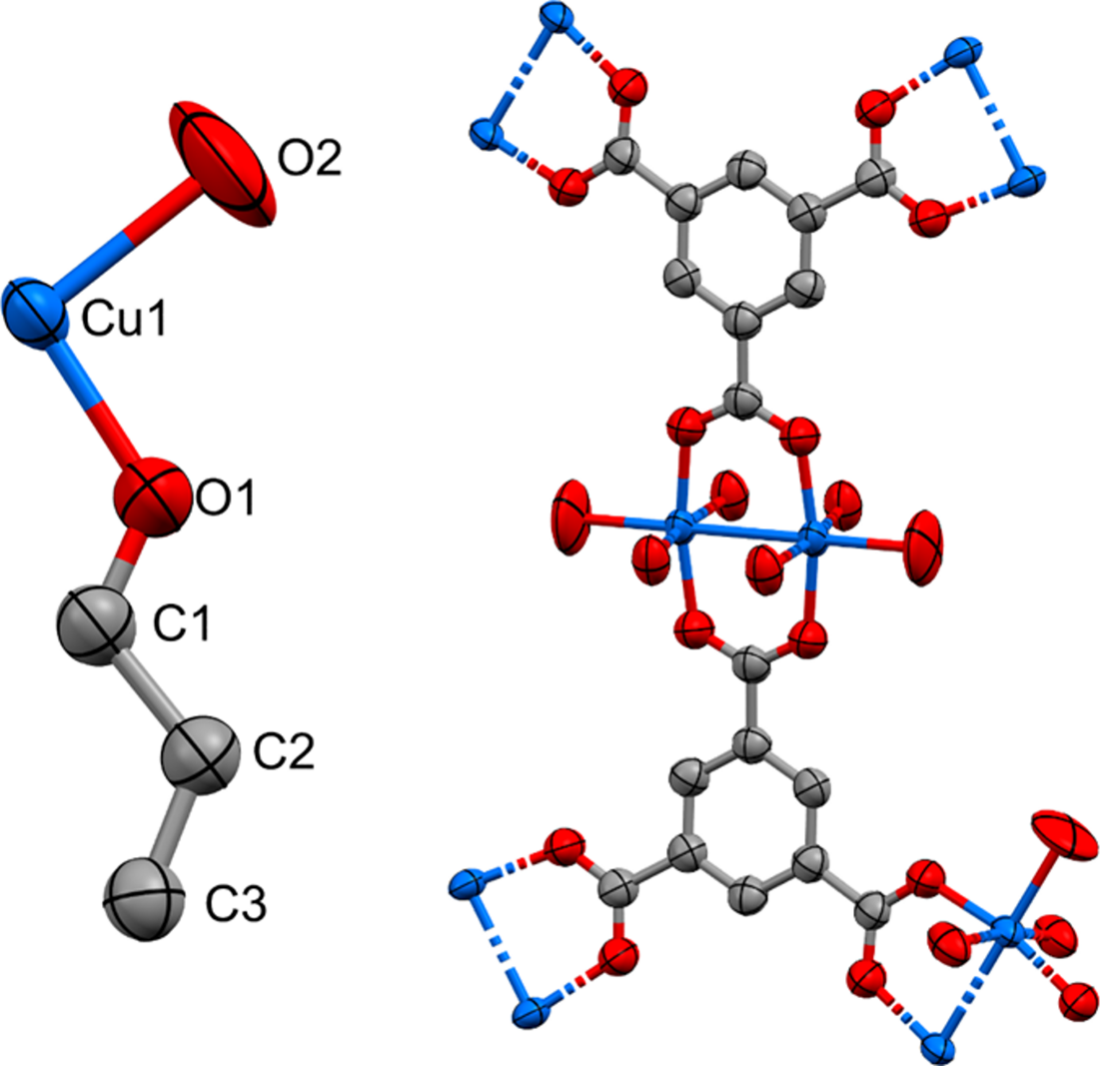
The crystal structure of **3** obtained by the PS method (left). The expanded polymeric structure is shown on the right. Displacement ellipsoids are drawn at the 50% probability level and H atoms have been omitted for clarity. Full crystal data are given in Table 3 ▸.

**Table 1:** Reduced X-ray crystallography data for sample **1** with crystals located using the DS and PS methods The structure is shown in Fig. 8 ▸ and full data are given in Table S2 of the supporting information.

	PS	DS
Total number of datasets	537	272
*I*/σ(*I*) lower filter	3.0	3.0
*R*_pim_ upper filter	0.2	0.2
Number datasets used	151	119
Phi centre positions (°)	0, 30, 60	0, 30, 60
Empirical formula	C_26_H_24_Cl_2_NiP_2_	C_26_H_24_Cl_2_NiP_2_
Temperature (K)	150	150
Space group	*P*2_1_/*c*	*P*2_1_/*c*
*a* (Å)	11.3742 (3)	11.3788 (2)
*b* (Å)	13.2950 (2)	13.29528 (17)
*c* (Å)	15.8494 (2)	15.8488(20
α (°)	90	90
β (°)	99.003 (2)	99.0149 (14)
γ (°)	90	90
*V* (Å^3^)	2367.22 (8)	2368.06 (7)
Completeness (%)	99.2	99.8
Limiting diffraction resolution (Å)	0.80	0.75
Reflections collected	71211	60273
Independent reflections	4790 (*R*_int_ = 0.1149, *R*_σ_ = 0.0493)	5500 (*R*_int_ = 0.0920, *R*_σ_ = 0.0394)
*R*_pim_	0.031	0.028
CC_1/2_	0.995	0.999
Data/restraints/parameters	4790/0/280	5500/0/280
Final *R* indexes [*I* ≥ 2σ(*I*)]	*R*_1_ = 0.0294, *wR*_2_ = 0.0719	*R*_1_ = 0.0297, *wR*_2_ = 0.0699
Final *R* indexes (all data)	*R*_1_ = 0.0398, *wR*_2_ = 0.0748	*R*_1_ = 0.0416, *wR*_2_ = 0.0732
Largest difference peak/hole (e Å^−3^)	0.32/−0.34	0.38/−0.31

**Table 2:** Reduced X-ray crystallography data for sample **2** with crystals located using the DS and PS methods The structure is shown in Fig. 9 ▸ and full data are given in Table S6 of the supporting information.

	PS	DS
Total number of datasets	296	443
*I*/σ lower filter	2	3
*R*_pim_ upper filter	0.2	0.2
Number datasets used	121	179
Phi centre positions (°)	0, 30, 60	0, 30, 60
Empirical formula	C_15_H_10_ClN_3_	C_15_H_10_ClN_3_
Temperature (K)	150	150
Space group	*Pna*2_1_	*Pna*2_1_
*a* (Å)	29.8128 (17)	29.815 (3)
*b* (Å)	3.8321 (2)	3.8313 (3)
*c* (Å)	10.6331 (4)	10.6315 (7)
α (°)	90	90
β (°)	90	90
γ (°)	90	90
*V* (Å^3^)	1214.79 (10)	1214.45 (17)
Completeness (%)	97.6	98.5
Limiting diffraction resolution (Å)	0.75	0.75
Reflections collected	33859	49789
Independent reflections	2944 (*R*_int_ = 0.1381, *R*_σ_ = 0.0563)	2969 (*R*_int_ = 0.1152, *R*_σ_ = 0.0394)
*R*_pim_	0.030	0.021
CC_1/2_	0.999	0.999
Data/restraints/parameters	2944/1/172	2969/1/172
Final *R* indexes [*I* ≥ 2σ(*I*)]	*R*_1_ = 0.0377, *wR*_2_ = 0.0811	*R*_1_ = 0.0312, *wR*_2_ = 0.0757
Final *R* indexes (all data)	*R*_1_ = 0.0482, *wR*_2_ = 0.0846	*R*_1_ = 0.0367, *wR*_2_ = 0.0780
Largest difference peak/hole (e Å^−3^)	0.17/−0.24	0.21/−0.21
Flack parameter	0.47 (9)	0.35 (7)

**Table 3:** Reduced X-ray crystallography data for sample **3** with crystals located using the DS and PS methods The structure is shown in Fig. 10 ▸ and full data are given in Table S10 of the supporting information.

	PS	DS
Total number of datasets	41	66
*I*/σ lower filter	3	3
*R*_pim_ upper filter	0.2	0.2
Number datasets used	22	63
Phi centre positions (°)	0, 30	0, 30
Empirical formula	C_12_H_4_Cu_2_O_10_	C_12_H_4_Cu_2_O_10_
Temperature (K)	150	150
Space group	*Fm* 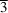 *m*	*Fm* 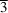 *m*
*a* (Å)	26.2857 (6)	26.2454 (3)
*b* (Å)	26.2857 (6)	26.2454 (3)
*c* (Å)	26.2857 (6)	26.2454 (3)
α (°)	90	90
β (°)	90	90
γ (°)	90	90
*V* (Å^3^)	18161.8 (12)	18078.4 (6)
Completeness (%)	98.3	97.6
Limiting diffraction resolution (Å)	0.75	0.75
Reflections collected	24456	69416
Independent reflections	1175 (*R*_int_ = 0.1266, *R*_σ_ = 0.0445)	1164 (*R*_int_ = 0.1429, *R*_σ_ = 0.0241)
*R*_pim_	0.032	0.021
CC_1/2_	0.994	0.993
Data/restraints/parameters	1175/0/36	1164/0/36
Final *R* indexes [*I* ≥ 2σ(*I*)]	*R*_1_ = 0.0345, *wR*_2_ = 0.0886	*R*_1_ = 0.0438, *wR*_2_ = 0.1208
Final *R* indexes (all data)	*R*_1_ = 0.0399, *wR*_2_ = 0.0903	*R*_1_ = 0.0468, *wR*_2_ = 0.1249
Largest difference peak/hole (e Å^−3^)	0.44/−0.34	0.38/−0.37

**Table 4:** Crystal size statistics as calculated from photometric data

Sample	**1**	**2**	**3**
Mean crystal dimension (µm)	6.8	7.2	18.4
Standard deviation of crystal dimension (µm)	5.0	8.4	8.3

## Data Availability

Data are available in the supporting information associated with this article, as well as from the CCDC. A GitHub repository containing the data and the image-processing software can also be found at https://github.com/DiamondLightSource/i19-PhotometrySerialCrystallography.
