# Supplementary material for: A picture is worth a thousand diffraction spots: using photometry and computer vision to perform rapid high-throughput sample-versatile serial crystallography
Source: IUCrJ. 2026 May 18;13(Pt 4):409–19. doi: 10.1107/S2052252526003568 (PMC13324597; doi:10.1107/S2052252526003568)
Supplement: Supplementary file 8 [file m-13-00409-sup8.pdf]

IUCrJ

Volume 13 (2026)

Supporting information for article:

A picture is worth a thousand diffraction spots: using photometry and computer vision to perform rapid high-throughput sample-versatile serial crystallography

Ben A. Coulson, Sam G. Lewis, Christian Orr, Lauren E. Hatcher, David R. Allan and Mark Warren

S1. A note on data quality metrics

$R_{int,}$ the internal R-value, is a measure of how the individual intensities (I) of equivalent reflections (hkl) compare with each other across a dataset. However, when considering datasets consisting of many merged collections across multiple crystals, large variations will arise in intensities as size and orientation varies across crystals. In general, as more redundant collections are incorporated into a serial dataset, R_{int} will tend to become larger, regardless of the overall data quality.

$$R_{int} = \frac{\Sigma_{hkl}\Sigma_i |I(hkl) - \langle I(hkl)\rangle|}{\Sigma_{hkl}\Sigma_i I(hkl)}$$

R_{pim} is similar to R_{int}, however also includes an additional factor that accounts for the multiplicity (or redundancy, n) of the data. This more correctly measures the uncertainty of the average reflection intensity in a high redundancy dataset, and more accurately reflects the additional precision that is obtained by a high redundancy multi-crystal dataset.

$$R_{pim} = \sqrt{\frac{1}{n-1}} \frac{\Sigma_{hkl}\Sigma_i |I(hkl) - \langle I(hkl)\rangle|}{\Sigma_{hkl}\Sigma_i I(hkl)}$$

S2. Physical Experimental Setup in EH2 at I19 (Diamond Light Source)

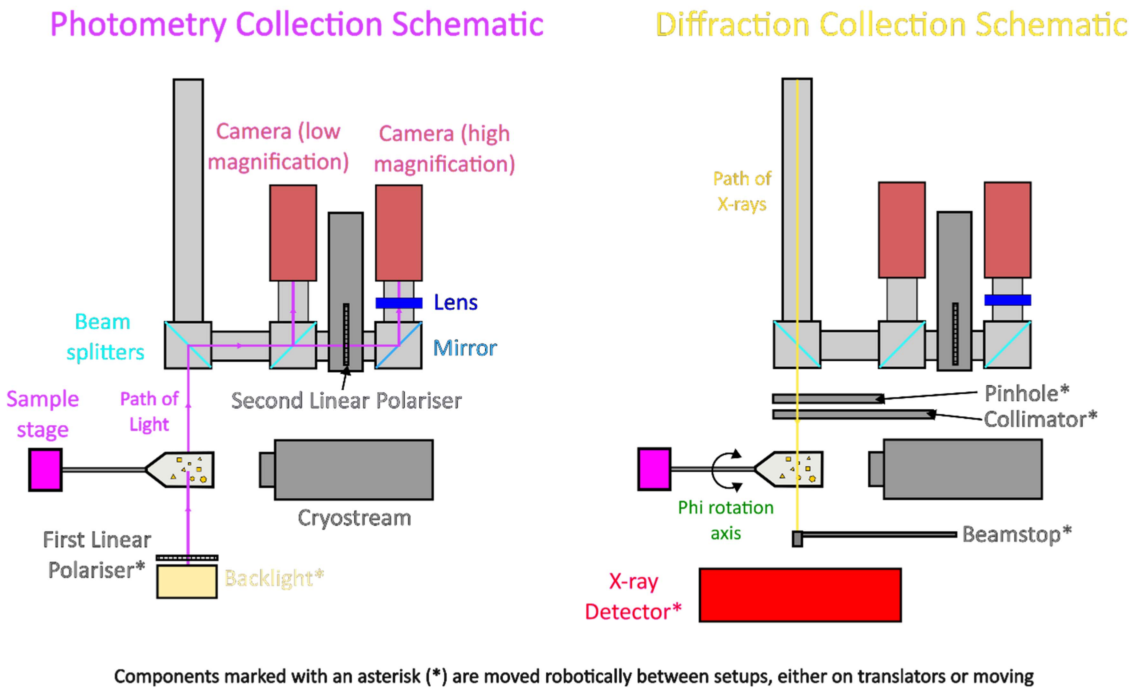

Figure S1 A schematic of the optical cameras and X-ray diffraction equipment set up in EH2 of I19 at Diamond.

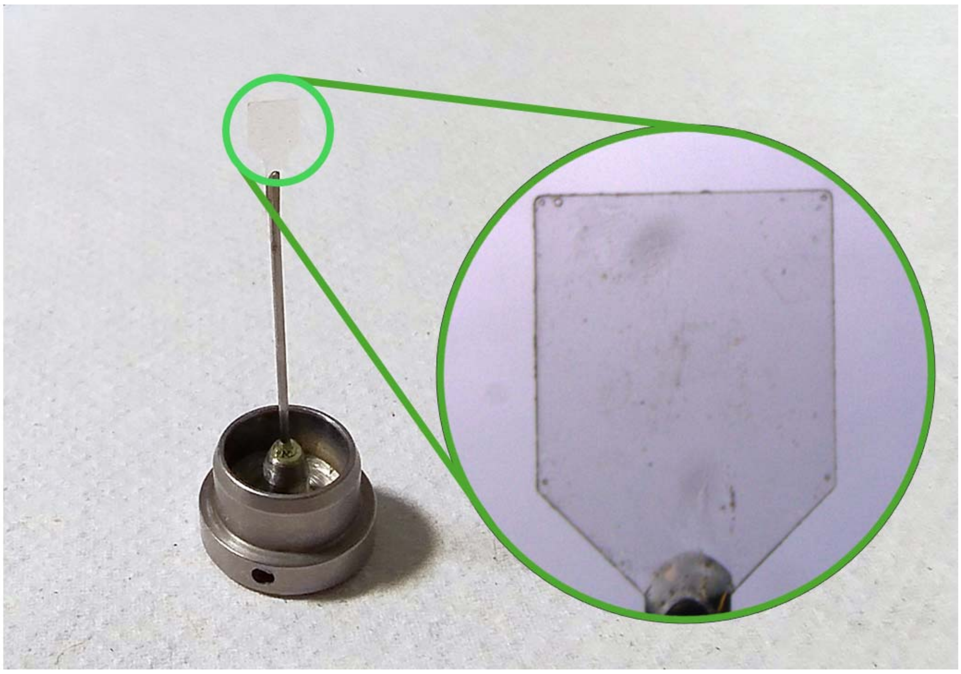

Figure S2 A substrate for serial data collections, inset, microscope image of an empty sample holder showing fiducial holes at each corner

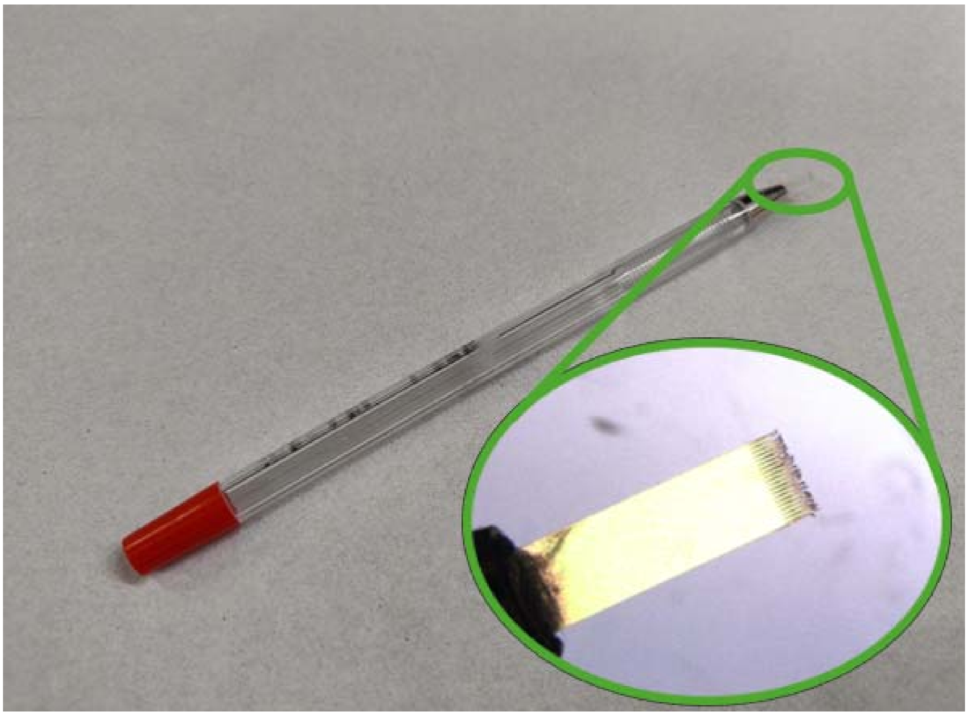

Figure S3 A 'crystal rake' used for manipulation of crystals across a substrate

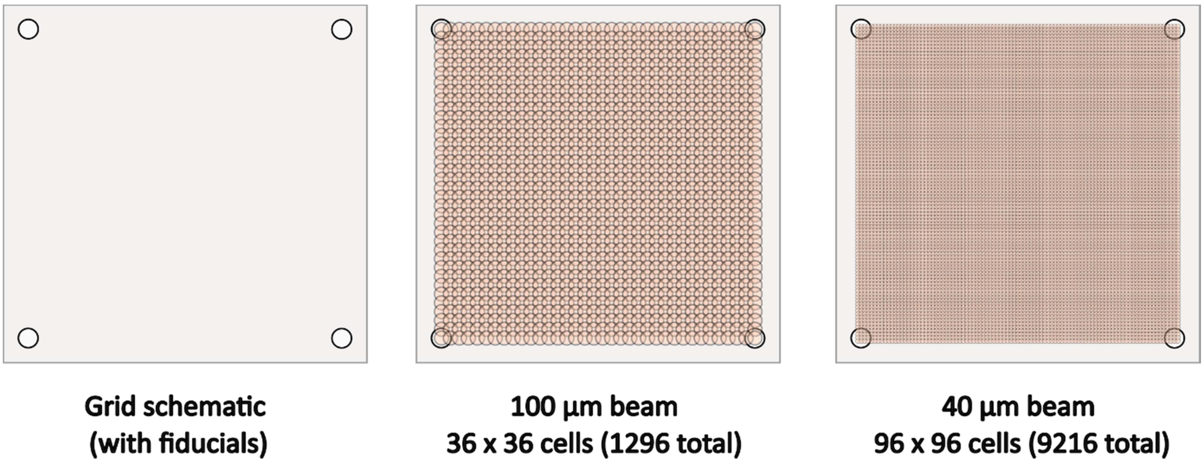

Figure S4 Schematics demonstrating how varying the beam size alters the number of cells required to cover the entire substrate when using the diffractive sweep method.

S3. Preferred Orientation of Crystals of 1

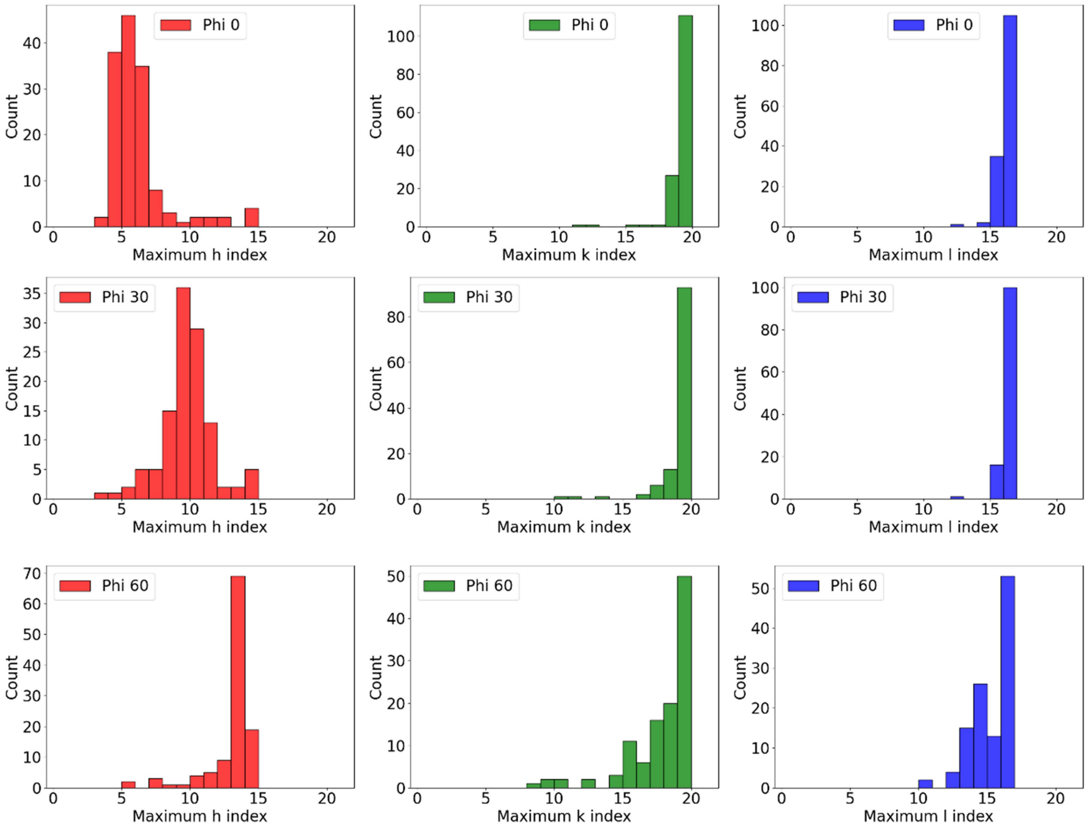

Figure S5 Maximum reachable miller indices obtained across a sample of 119 crystals of **1** identified using the diffractive sweep method, comparing starting φ angles of 0°, 30° and 60°. Clear preferred orientation is shown.

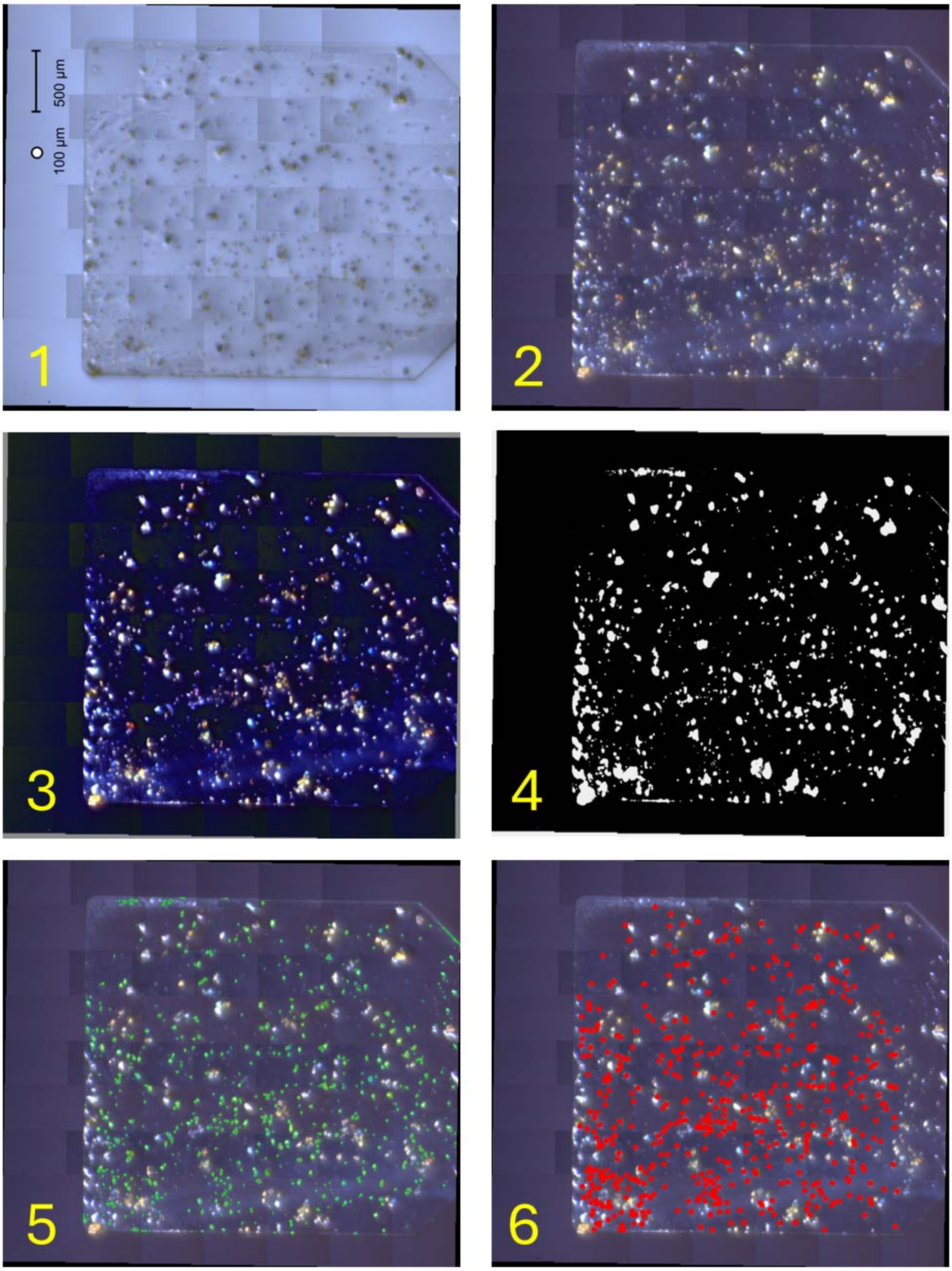

Figure S6 Individual image processing steps taken for **1** (see table S1). The initial microscopy image of the sample with scale bar (1), the sample through crossed linear polarisers (2), after contrast and brightness corrections (3), after binarization (4), visualisation of the contours located by OpenCV (5), and final locations of crystals overlaid on sample (6). See also Table S1.

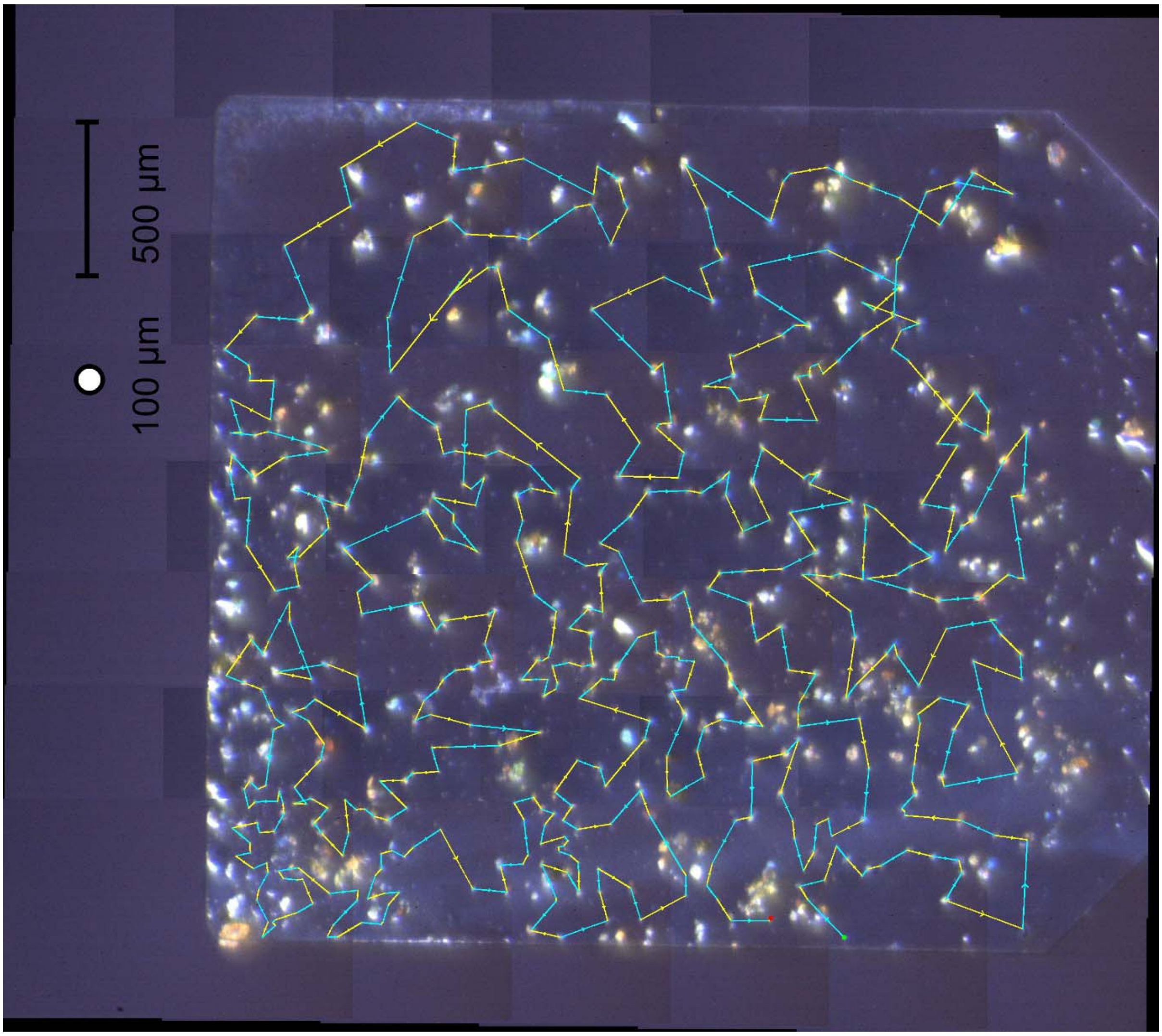

Figure S7 Final optimised crystal locations and optimised pathway (green to red) for **1** across 537 identified crystals. Pixel positions from this image were related to motor positions and used for collection. Optimised path was 11% the length of a raster pathway. Each blue and yellow segment represents a movement of the X-ray beam relative to the sample between crystal sites, with a 5° data collection occurring at each vertex. There is no difference between blue and yellow movements: the variation in colour is simply a guide to the eye. Arrows in middle of path indicate the direction of travel of the X-ray beam relative to the sample. Note that in reality, the X-ray beam is static and the entire sample is moving in reverse direction of the arrows.

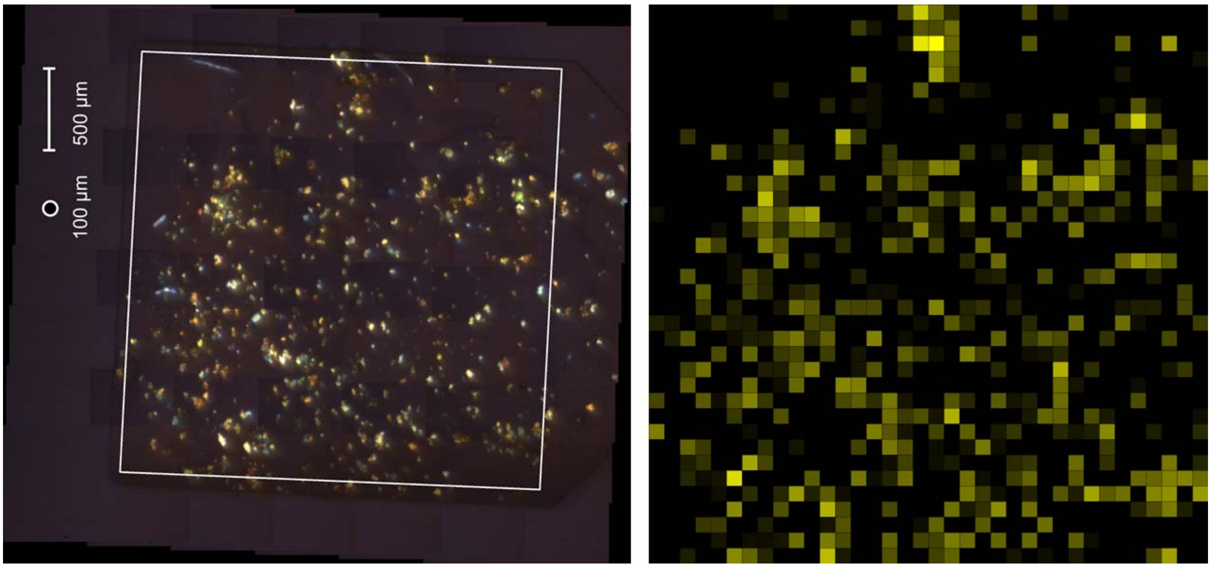

Figure S8 Photograph of substrate loaded with **1** (left), compared to ‘diffraction map’ (right) where pixel brightness corresponds to number of diffraction spots at that cell. The area on the photograph corresponding to the collection area is denoted by the white box.

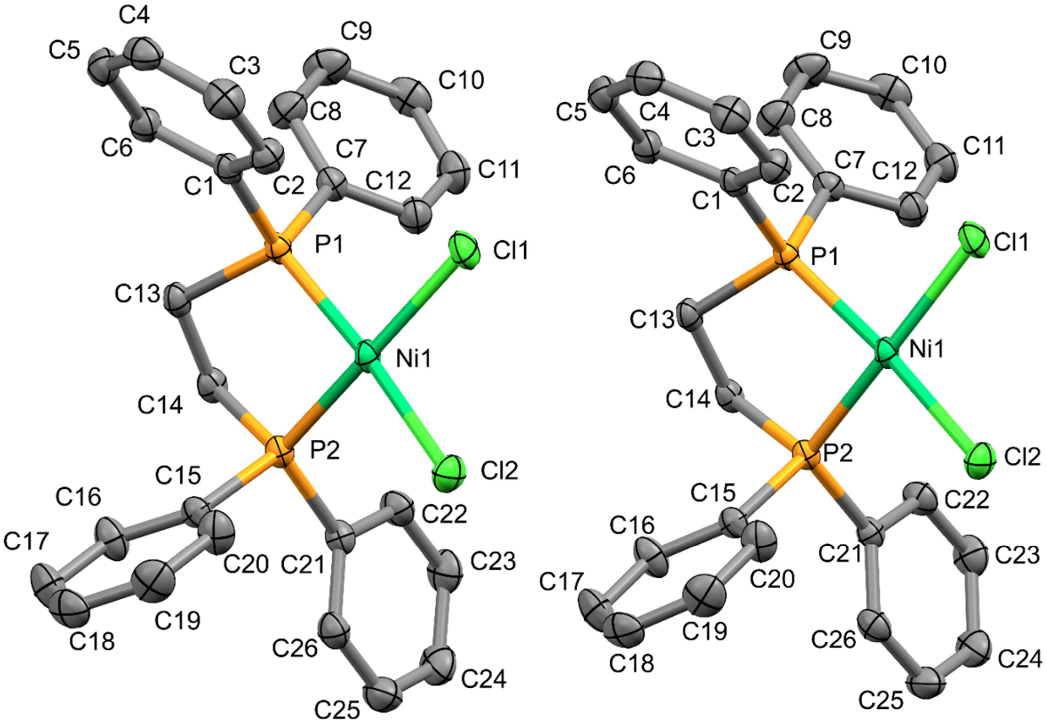

Figure S9 Crystal structures of **1** with crystal located by the PS method (left) and DS method (right). Ellipsoids drawn at 50% probability, hydrogen atoms omitted for clarity.

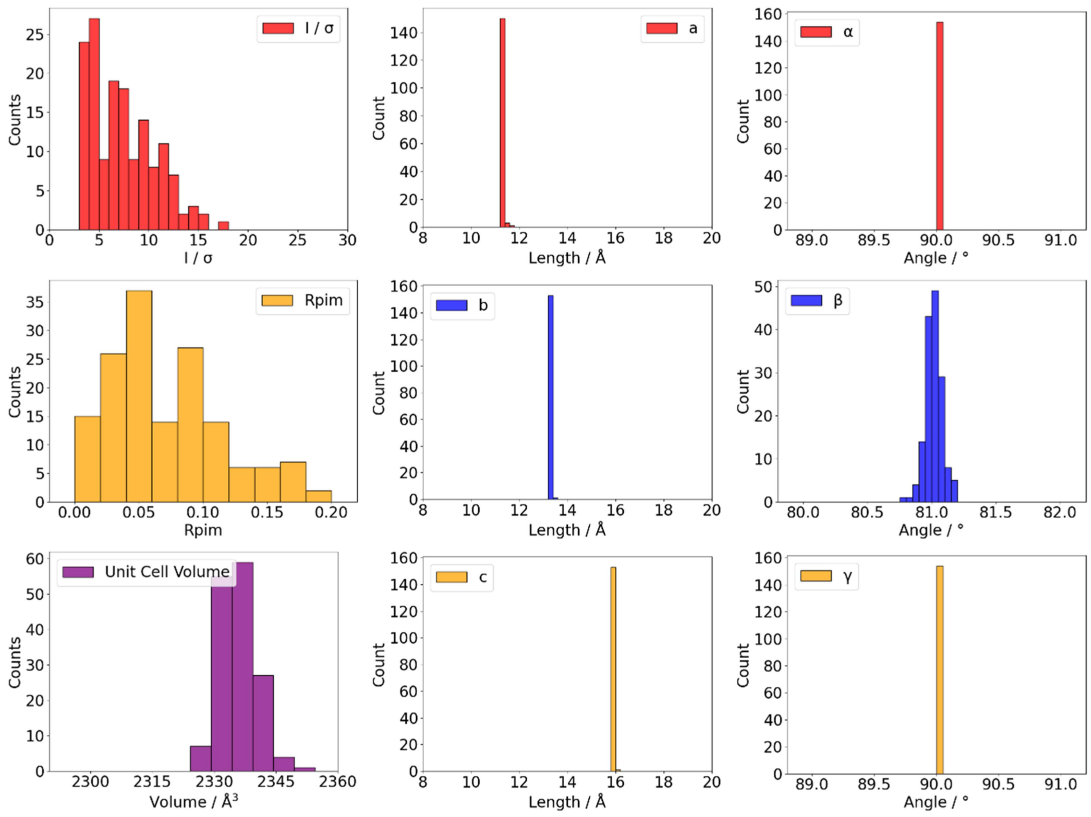

Figure S10 Statistics from 151 crystals of **1** identified by photometric selection (PS)

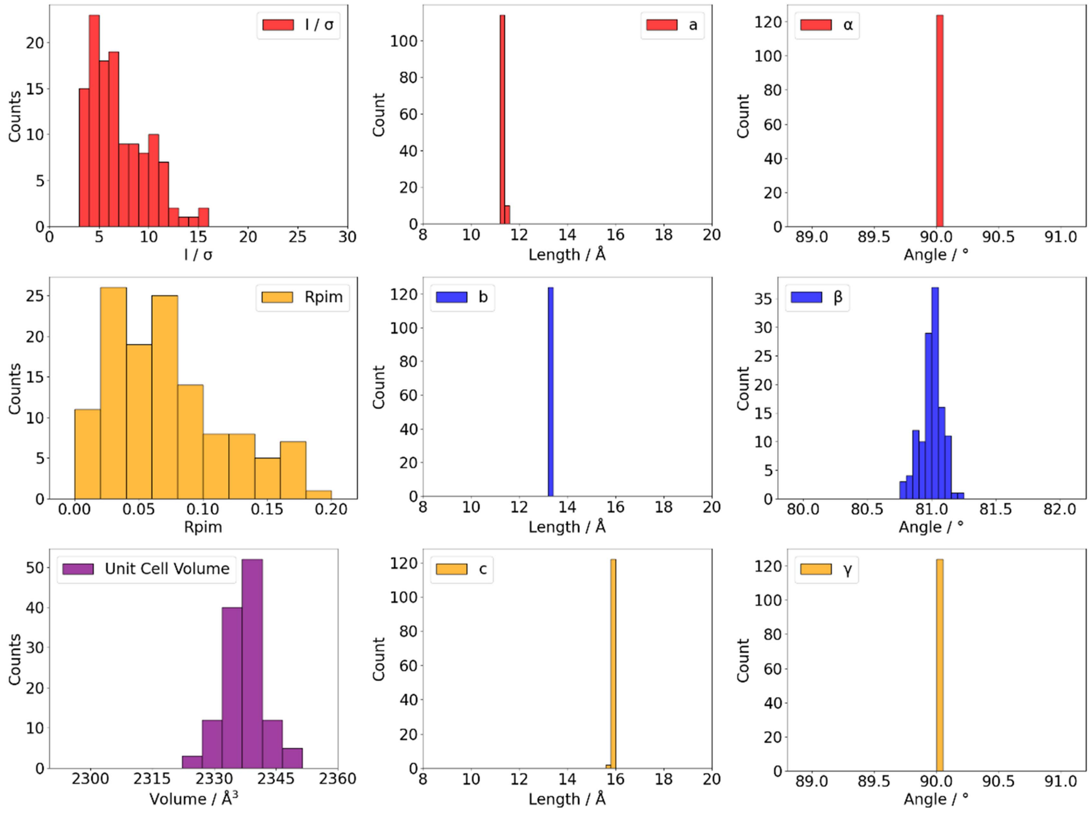

Figure S11 Statistics from 119 crystals of **1** identified by diffractive sweep method (DS)

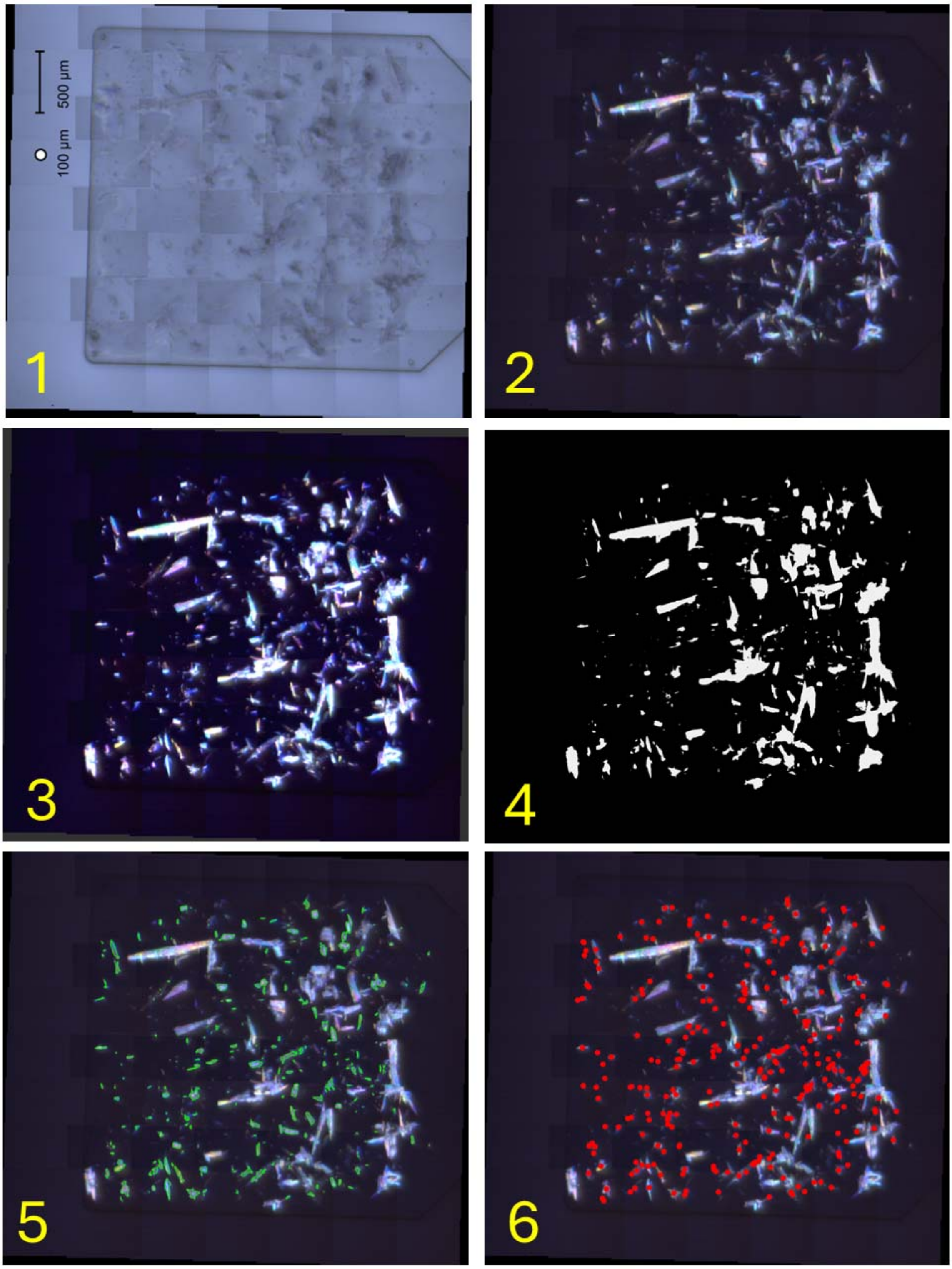

Figure S12 Individual image processing steps taken for 2 (see table S1). The initial microscopy image of the sample with scale bar (1), the sample through crossed linear polarisers (2), after contrast and brightness corrections (3), after binarization (4), visualisation of the contours located by OpenCV (5), and final locations of crystals overlaid on sample (6).

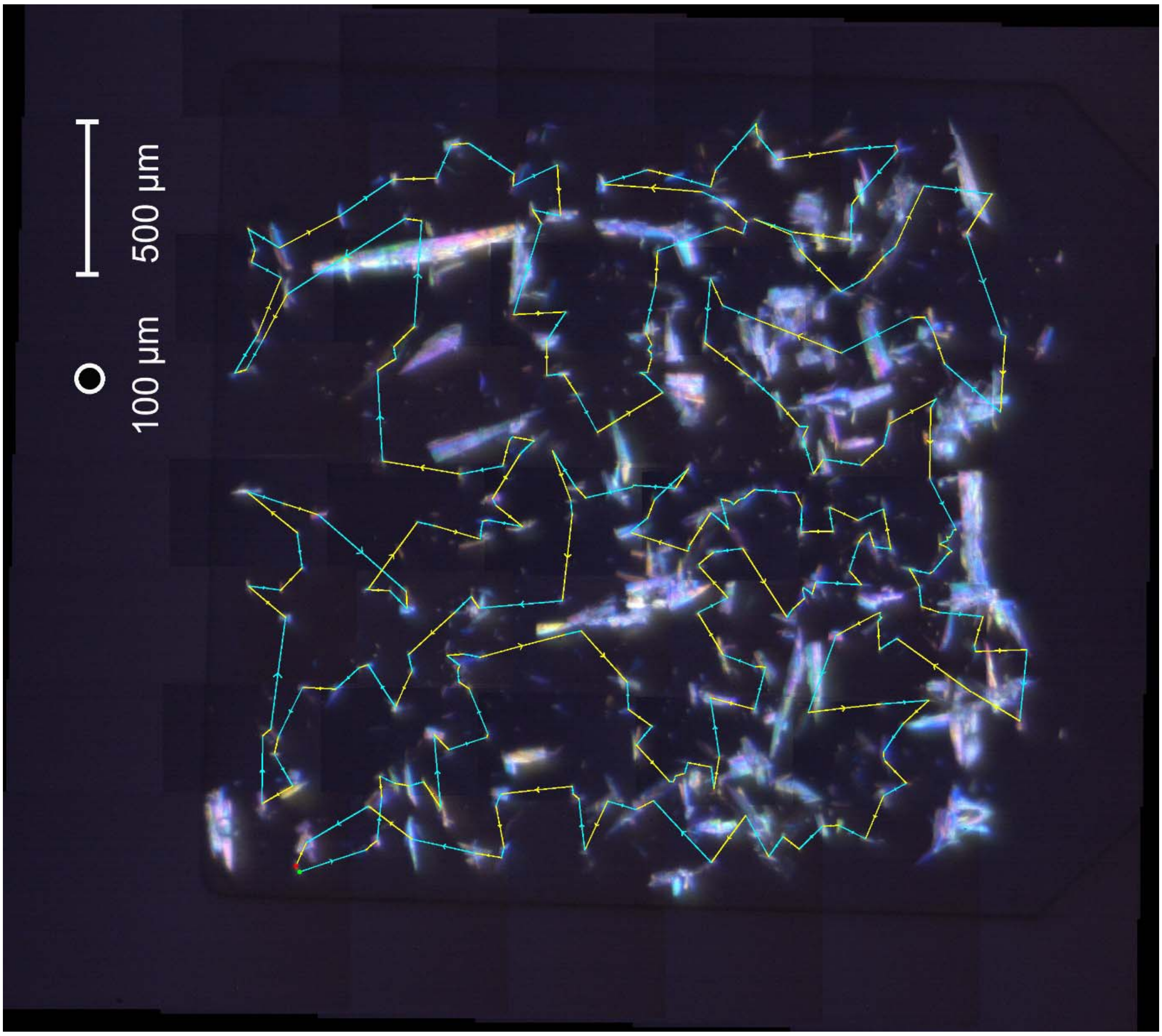

Figure S13 Final optimised crystal locations and optimised pathway (green to red) for **2** across 296 identified crystals. Pixel positions from this image were related to motor positions and used for collection. Optimised path was 15% the length of a raster pathway. . Each blue and yellow segment represents a movement of the X-ray beam relative to the sample between crystal sites, with a 5° data collection occurring at each vertex. There is no difference between blue and yellow movements: the variation in colour is simply a guide to the eye. Arrows in middle of path indicate the direction of travel of the X-ray beam relative to the sample. Note that in reality, the X-ray beam is static and the entire sample is moving in reverse direction of the arrows.

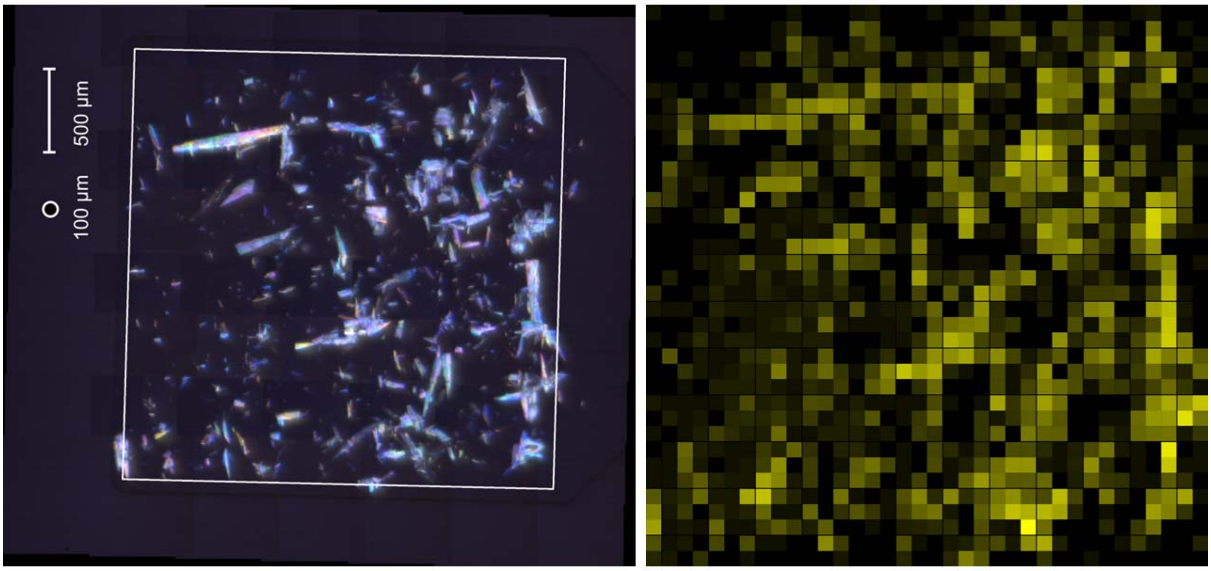

Figure S14 Photograph of substrate loaded with **2** (left), compared to 'diffraction map' (right) where pixel brightness corresponds to number of diffraction spots at that cell. The area on the photograph corresponding to the collection area is denoted by the white box.

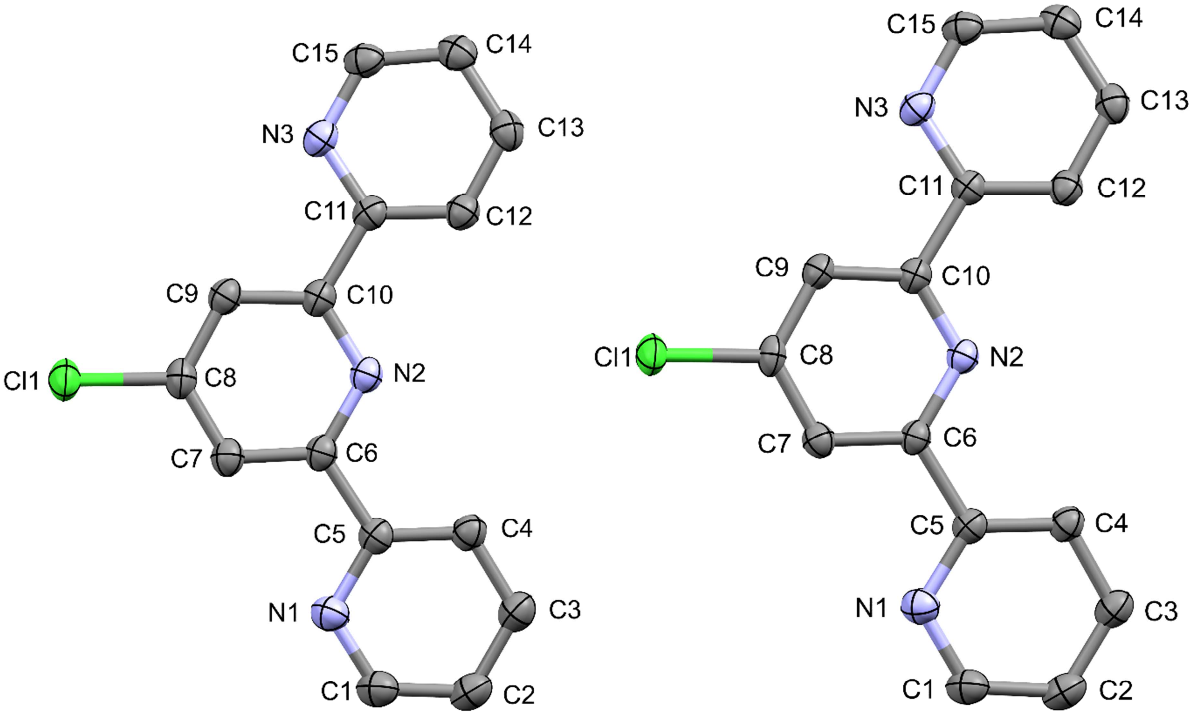

Figure S15 Crystal structures of **2** with crystal located by the PS method (left) and DS method (right). Ellipsoids drawn at 50% probability, hydrogen atoms omitted for clarity.

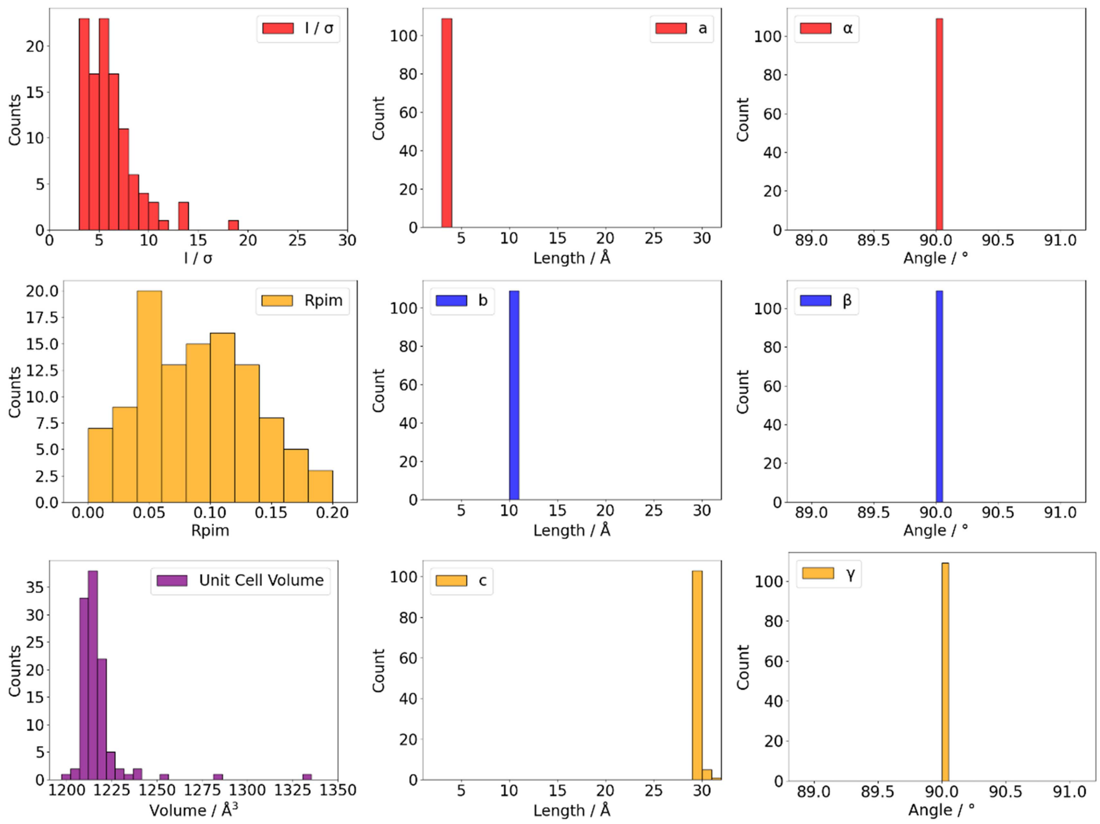

Figure S16 Statistics from 121 crystals of **2** identified by photometric selection (PS)

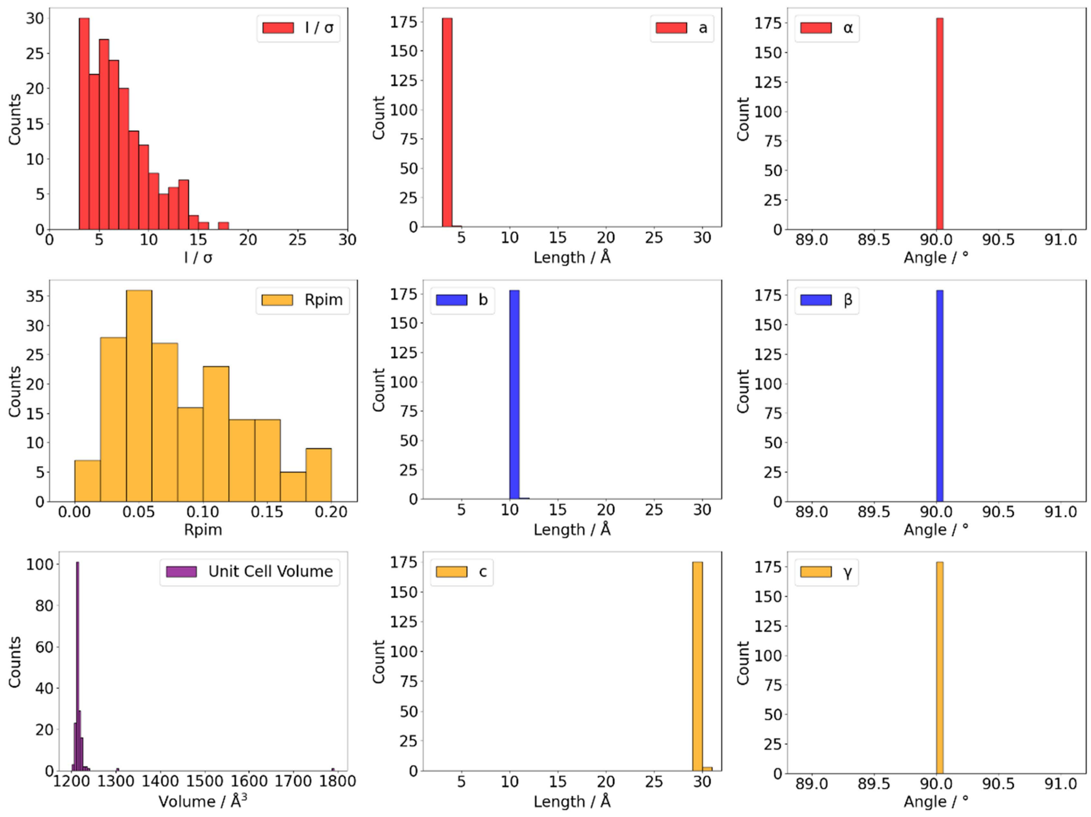

Figure S17 Statistics from 179 crystals of **2** identified by diffractive sweep method (DS)

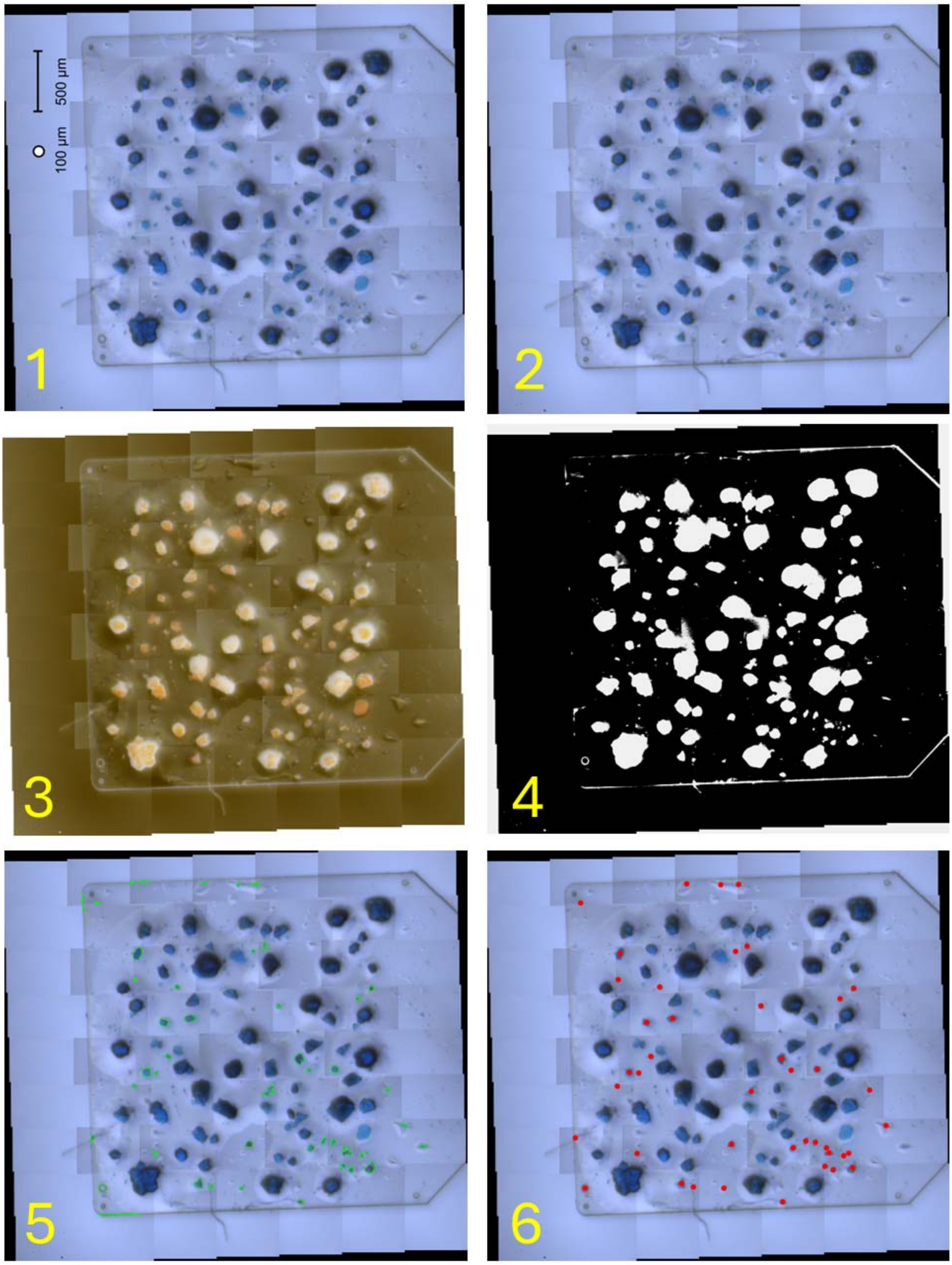

Figure S18 Individual image processing steps taken for **3** (see table S9). The initial microscopy image of the sample with scale bar (1), the sample through crossed linear polarisers (2), after contrast and brightness corrections and colour inversion (3), after binarization (4), visualisation of the contours located by OpenCV (5), and final locations of crystals overlaid on sample (6).

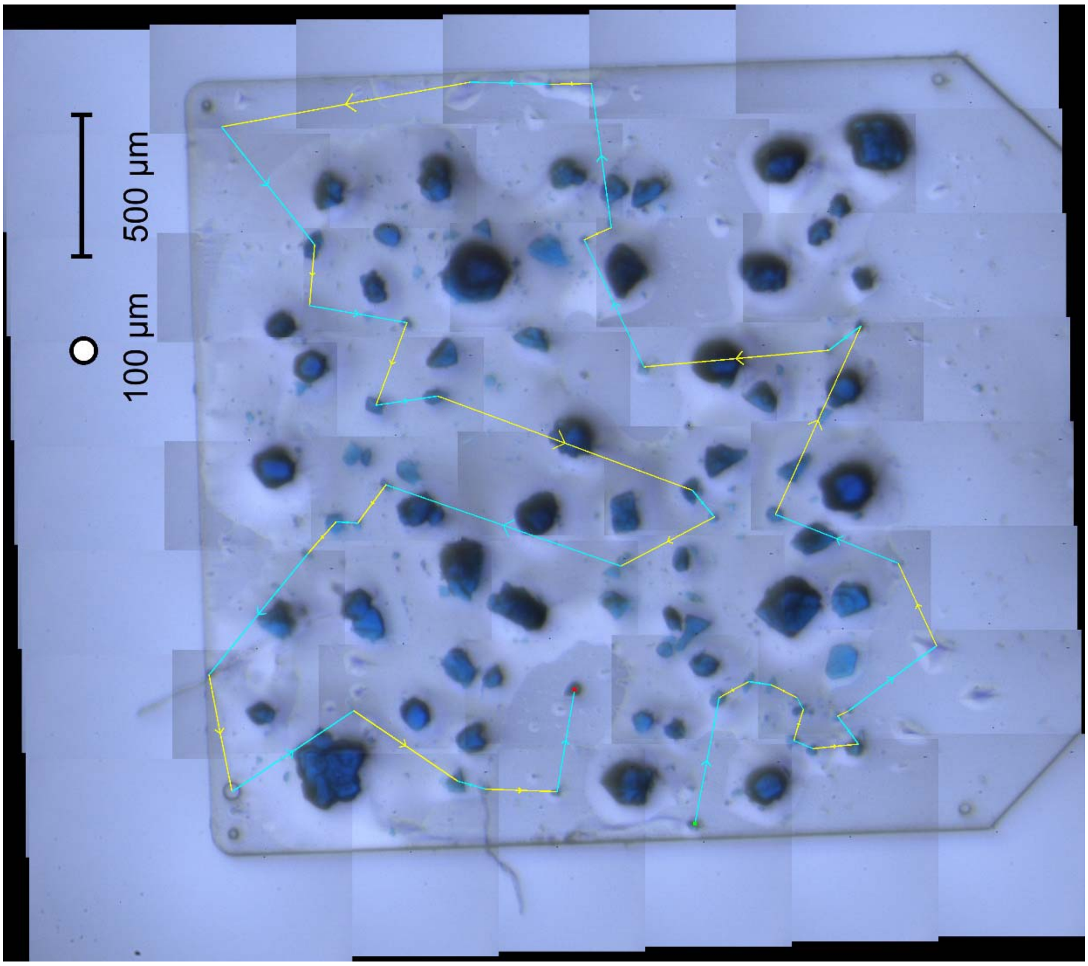

Figure S19 Final optimised crystal locations and optimised pathway (green to red) for **3** across 41 identified crystals. Pixel positions from this image were related to motor positions and used for collection. Optimised path was 42% the length of a raster pathway. . Each blue and yellow segment represents a movement of the X-ray beam relative to the sample between crystal sites, with a 5° data collection occurring at each vertex. There is no difference between blue and yellow movements: the variation in colour is simply a guide to the eye. Arrows in middle of path indicate the direction of travel of the X-ray beam relative to the sample. Note that in reality, the X-ray beam is static and the entire sample is moving in reverse direction of the arrows.

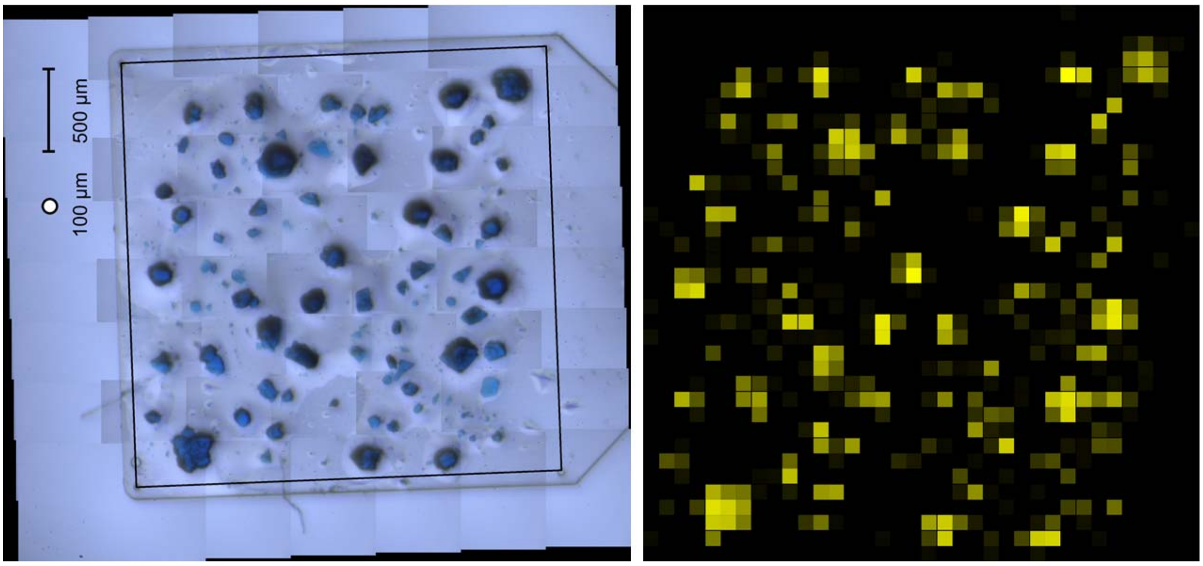

Figure S20 Photograph of substrate loaded with **3** (left), compared to 'diffraction map' (right) where pixel brightness corresponds to number of diffraction spots at that cell. The area on the photograph corresponding to the collection area is denoted by the white box.

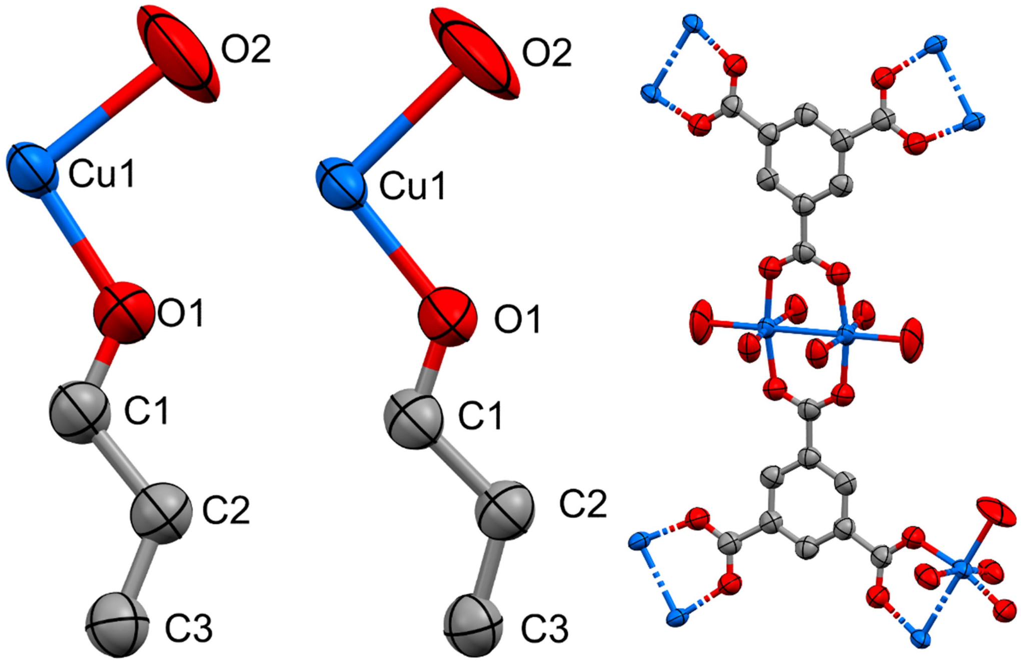

Figure S21 Crystal structures of 2 with crystal located by the PS method (left) and DS method (centre). See expanded structure on right for polymeric structure. Ellipsoids drawn at 50% probability, hydrogen atoms omitted for clarity.

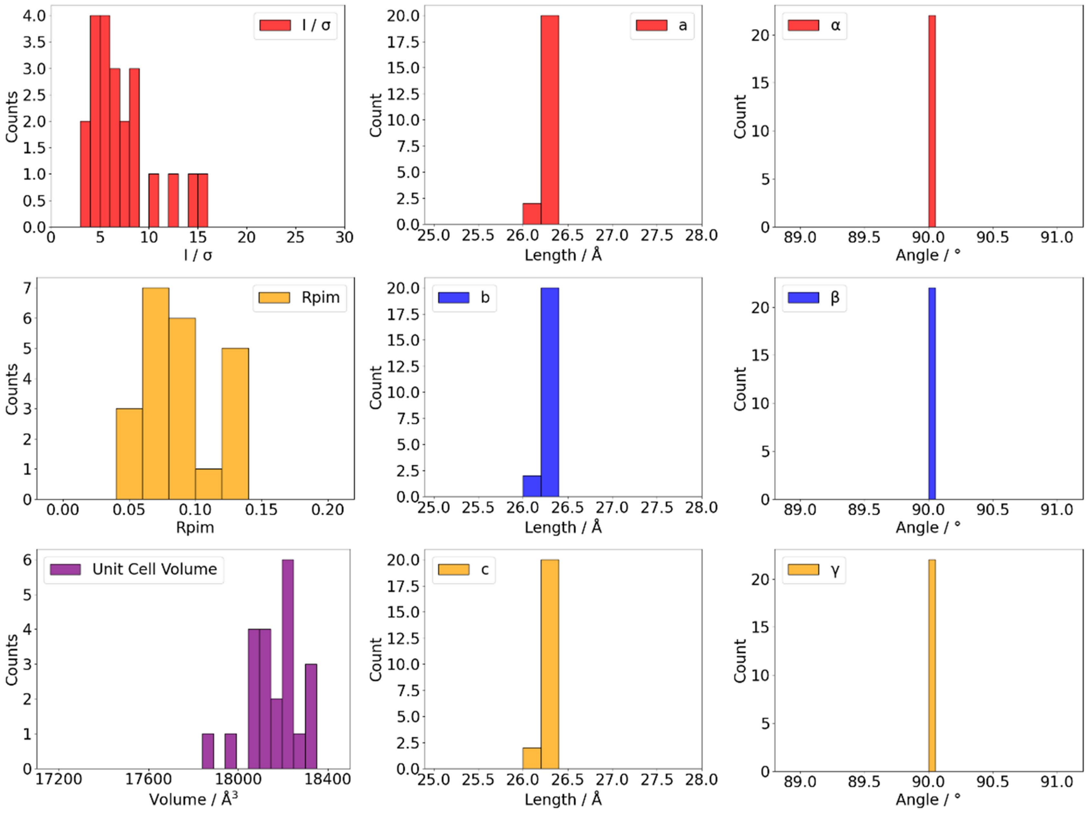

Figure S22 Statistics from 22 crystals of **3** identified by photometric selection (PS)

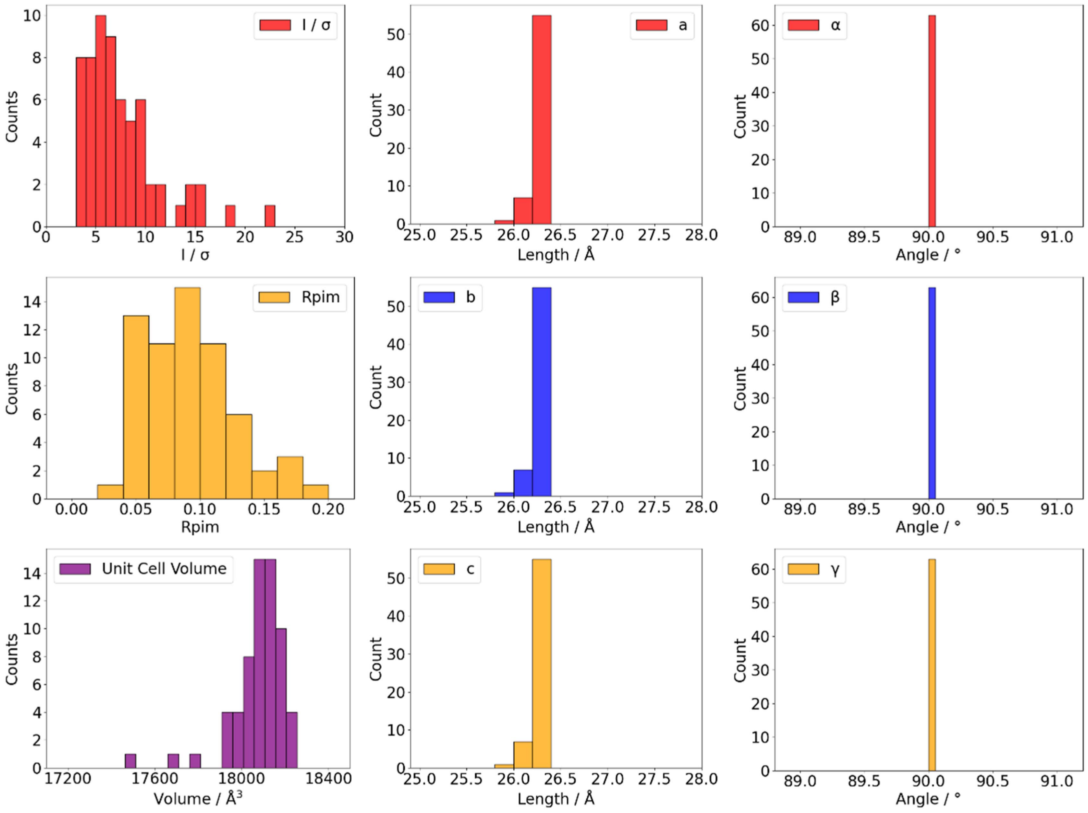

Figure S23 Statistics from 63 crystals of **3** identified by diffractive sweep method (DS)

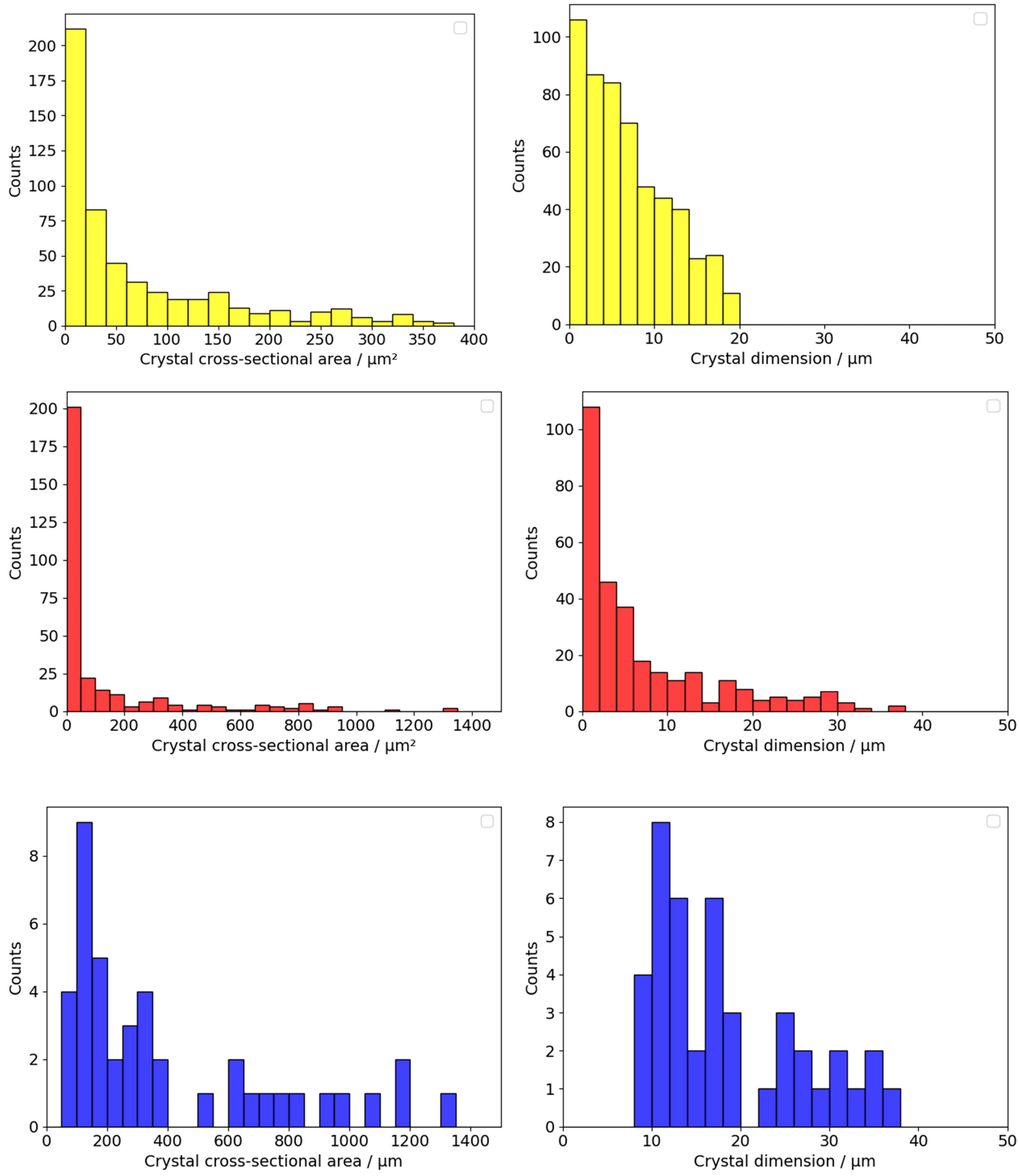

Figure S24 Histograms demonstrating crystal size distributions across the substate for **1** (top, yellow), **2** (middle, red) and **3** (bottom, blue). Sizes are collected from the OpenCV contours objects, which have a cross-sectional area expressed in pixels. By applying a known scaling factor of 0.68 µm / pixel these cross sectional areas are converted from pixels into micrometres-squared. Crystal dimension, which is characterised as the mean distance between opposite crystal faces, is estimated by taking the square root of the cross-sectional areas. See Table S13 for further statistics.

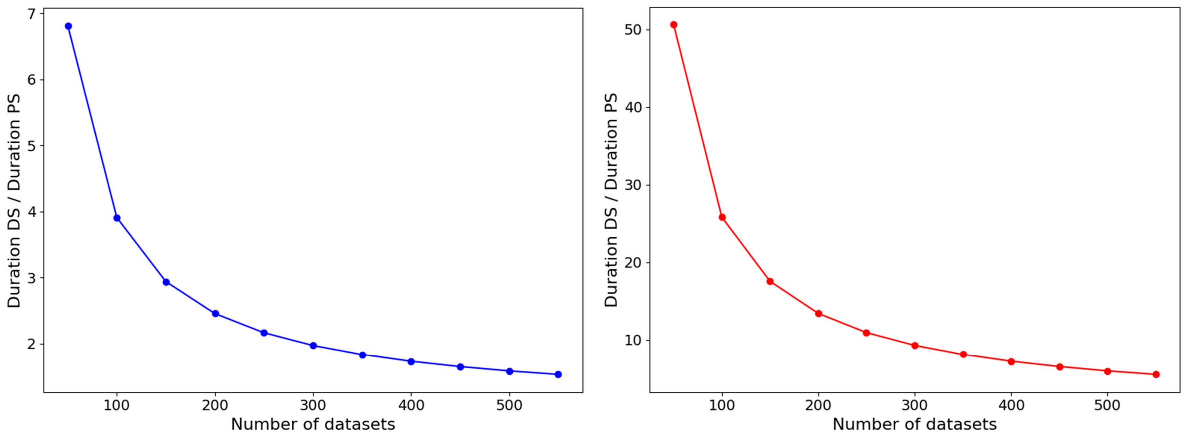

Figure S25 Plot of number of collection datasets against the estimated ratio of the duration of a PS run to a DS run when using a 100 micron beam (left) and 40 micron beam (right). For example, performing 50 data collections on a sample using the PS method would be almost 7 times faster than the same 50 collections using the DS method. The difference in timings arises from the initial time cost of collecting the grid scan. As the beam size gets smaller, scanning the grid takes longer, accounting for the marked increase in speed of PS compared to DS when moving to a 40 micron beam.

Table S1 Summary of image processing steps taken in Figure S6

Image	Description
1	Raw stitched image with scale bar and beam size indicator.
2	Polarising filter and light source applied orthogonal to each other to display birefringence
3	Contrast adjusted (2.0), brightness adjusted (-140)
4	Image is binarised according to an upper threshold of 240, a lower threshold of 80, and using a gaussian blur of 3
5	Contours of crystals are located using OpenCV and filtered out with an upper path size limit of 800 square pixels and a lower limit of 0 square pixels. Green contours are overlaid onto original polarised image
6	Crystal centres are overlaid onto original polarised image (red dots represent found crystal)

Table S2 X-ray crystallography data for **1** with crystals located using the DS and PS methods

	**PS**	**DS**
Total Number of datasets	537	272
I/σ(I) Lower Filter	3.0	3.0
R_{pim} Upper Filter	0.2	0.2
Number datasets Used	151	119
Phi Centre Positions / °	0, 30, 60	0, 30, 60
Empirical Formula	$C_{26}H_{24}Cl_2NiP_2$	$C_{26}H_{24}Cl_2NiP_2$
Formula Weight	528.00	528.00
Temperature / K	150	150
Crystal System	monoclinic	monoclinic
Space group	$P2_1/c$	$P2_1/c$
a / Å	11.3742(3)	11.3788(2)
b / Å	13.2950(2)	13.29528(17)
c / Å	15.8494(2)	15.8488(20
α / °	90	90
β / °	99.003(2)	99.0149(14)
γ / °	90	90
Volume / $Å^3$	2367.22(8)	2368.06(7)
Z, Z'	4, 1	4, 1
ρ_{calc} / g/cm^3	1.482	1.481
μ / mm^{-1}	0.414	0.414
F(000)	1088.0	1088.0
Radiation λ / Å	0.4859	0.4859
Index Values	$-14 \le h \le 14$, $-16 \le k \le 16$, $-19 \le l \le 19$	$-15 \le h \le 15$, $-17 \le k \le 17$, $-20 \le l \le 20$
Completeness / %	99.2	99.8
Reflections Collected	77162	60273
Independent Reflections	4790 [R_{int} = 0.1149,	5500 [R_{int} = 0.0920,

	R_{sigma} = 0.0493]	R_{sigma} = 0.0394]
R_{pim}	0.031	0.028
CC ½	0.995	0.999
Data/Restraints/Parameters	4790/0/280	5500/0/280
Goodness-of-fit on F^2	1.055	1.035
Final R indexes [I >= 2σ (I)]	R_1 = 0.0294, wR_2 = 0.0719	R_1 = 0.0297, wR_2 = 0.0699
Final R indexes [all data]	R_1 = 0.0398, wR_2 = 0.0748	R_1 = 0.0416, wR_2 = 0.0732
Largest diff. peak/hole e $Å^{-3}$	0.32 / -0.34	0.38/-0.31

Table S3 Statistics of 151 crystals of **1** identified by photometric selection method (PS)

Metric	Mean	Standard Deviation	Mode	Median
I/σ(I)	7.4	3.2	4.7	6.9
R_{pim}	0.071	0.044	0.038	0.059
$CC_{1/2}$	0.982	0.034	0.998	0.994
Volume / $Å^3$	2336.7	12.5	2336.1	2336.2
α / °	90	0	90	90
β / °	81.014	0.064	81.060	81.011
γ / °	90	0	90	90
a / Å	11.364	0.0272	11.345	11.361
b / Å	13.298	0.0219	13.284	13.296
c / Å	15.850	0.0262	15.843	15.849

Table S4 Statistics of 119 crystals of **1** identified by diffractive sweep method (DS)

Metric	Mean	Standard Deviation	Mode	Median
I/σ(I)	6.9	2.9	7.2	6.1
R_{pim}	0.072	0.044	0.057	0.062
$CC_{1/2}$	0.959	0.184	0.998	0.992
Volume / $Å^3$	2337.4	4.8	2338.5	2337.4
α / °	90	0	90	90
β / °	90	0	90	90
γ / °	80.993	0.0832	80.960	81.002
a / Å	11.372	0.0198	11.369	11.373
b / Å	13.295	0.0132	13.288	13.296
c / Å	15.484	0.0181	15.828	15.849

Table S5 Summary of image processing steps taken for **2** in Figure S12

Step	Description
1	Polarising filter and light source applied orthogonal to each other to display birefringence

2	Contrast adjusted (2.0), brightness adjusted (-50)
3	Image is binarised according to an upper threshold of 240, a lower threshold of 120, and using a gaussian blur of 1
4	Contours of crystals are located using OpenCV and filtered out with an upper path size limit of 3000 square pixels and a lower limit of 0 square pixels. Green contours are overlaid onto original polarised image
5	Crystal centres are overlaid onto polarised image (red dots represent found crystal)

Table S6 X-ray crystallography data for **2** with crystals located using the DS and PS methods

	**PS**	**DS**
Total Number of datasets	296	443
I/sigma Lower Filter	2	3
R_{pim} Upper Filter	0.2	0.2
Number datasets Used	121	179
Phi Centre Positions / °	0, 30, 60	0, 30, 60
Empirical Formula	$C_{15}H_{10}ClN_3$	$C_{15}H_{10}ClN_3$
Formula Weight	267.71	267.71
Temperature / K	150	150
Crystal System	Orthorhombic	Orthorhombic
Space group	$Pna2_1$	$Pna2_1$
a / Å	29.8128(17)	29.815(3)
b / Å	3.8321(2)	3.8313(3)
c / Å	10.6331(4)	10.6315(7)
α / °	90	90
β / °	90	90
γ / °	90	90
Volume / $Å^3$	1214.79(10)	1214.45(17)
Z, Z’	4,1	4,1
ρ_{calc} / g/cm^3	1.464	1.464
μ / mm^{-1}	0.115	0.115

F(000)	552.0	552.0
Radiation λ / Å	0.4859	0.4859
Index Values	-39 ≤ h ≤ 39, -5 ≤ k ≤ 5,	-39 ≤ h ≤ 39, -5 ≤ k ≤ 5,
	-14 ≤ l ≤ 14	-14 ≤ l ≤ 14
Completeness / %	97.6	98.5
Reflections Collected	33859	49789
Independent Reflections	2944 [R_{int} = 0.1381,	2969 [R_{int} = 0.1152,
	R_{sigma} = 0.0563]	R_{sigma} = 0.0394]
R_{pim}	0.030	0.021
CC ½	0.999	0.999
Data/Restraints/Parameters	2944/1/172	2969/1/172
Goodness-of-fit on F^2	0.968	1.038
Final R indexes [I >= 2σ (I)]	R_1 = 0.0377, wR_2 = 0.0811	R_1 = 0.0312, wR_2 = 0.0757
Final R indexes [all data]	R_1 = 0.0482, wR_2 = 0.0846	R_1 = 0.0367, wR_2 = 0.0780
Largest diff. peak/hole e $Å^{-3}$	0.17/-0.24	0.21/-0.21
Flack Parameter	0.47(9)	0.35(7)

Table S7 Statistics of 121 crystals of **2** identified by photometric selection method (PS)

Metric	Mean	Standard Deviation	Mode	Median
I/σ(I)	6.0	2.6	5.4	5.4
R_{pim}	0.089	0.046	0.057	0.083
$CC_{1/2}$	0.965	0.0603	0.998	0.998
Volume / Å^3	1217.2	14.9	1211.6	1213.9
α / °	90	0	90	90
β / °	90	0	90	90
γ / °	90	0	90	90
a / Å	3.833	0.0129	3.833	3.831
b / Å	10.636	0.0325	10.640	10.630
c / Å	29.854	0.182	29.820	29.820

Table S8 Statistics of 179 crystals of **2** identified by diffractive sweep method (DS)

Metric	Mean	Standard Deviation	Mode	Median
I/σ(I)	7.0	3.0	6.4	6.3
R_{pim}	0.084	0.048	0.050	0.074
$CC_{1/2}$	0.975	0.0387	0.999	0.990
Volume / Å^3	1218.4	43.9	1208.2	1214.0
α / °	90	0	90	90
β / °	90	0	90	90
γ / °	90	0	90	90
a / Å	3.835	3.832	3.831	0.0394
b / Å	10.640	10.632	10.632	0.0785
c / Å	29.853	29.800	29.807	0.428

Table S9 Summary of image processing steps taken for **3** in Figure S18

Step	Description
1	Contrast adjusted (1.0), brightness adjusted (1)

2	Cubic samples do not display birefringence and so in lieu of this, the image colours are inverted
3	Image is binarised according to an upper threshold of 240, a lower threshold of 120, and using a gaussian blur of 1
4	Contours of crystals are located using OpenCV and filtered out with an upper path size limit of 3000 square pixels and a lower limit of 150 square pixels. Green contours are overlaid onto original polarised image
5	Crystal centres are overlaid onto polarised image (red dots represent found crystal)

Table S10 X-ray crystallography data for sample **3** with crystals located using the DS and PS methods

	**PS**	**DS**
Total Number of datasets	41	66
I/sigma Lower Filter	3	3
R_{pim} Upper Filter	0.2	0.2
Number datasets Used	22	63
Phi Centre Positions / °	0, 30	0, 30
Empirical Formula	$C_{12}H_4Cu_2O_{10}$	$C_{12}H_4Cu_2O_{10}$
Formula Weight	435.23	435.23
Temperature / K	150	150
Crystal System	Cubic	Cubic
Space group	*Fm*-3*m*	*Fm*-3*m*
a / Å	26.2857(6)	26.2454(3)
b / Å	26.2857(6)	26.2454(3)
c / Å	26.2857(6)	26.2454(3)
α / °	90	90
β / °	90	90
γ / °	90	90
Volume / $Å^3$	18161.8(12)	18078.4(6)
Z, Z’	24,0.125	24,0.125

ρ_{calc} / g/cm^3	0.955	0.959
μ / mm^{-1}	0.498	0.500
F(000)	5136.0	5136.0
Radiation λ / Å	0.4859	0.4859
Index Values	-34 ≤ h ≤ 35, -35 ≤ k ≤ 34, -34 ≤ l ≤ 34	-34 ≤ h ≤ 34, -34 ≤ k ≤ 34, -34 ≤ l ≤ 34
Completeness / %	98.6	98.2
Reflections Collected	24456	69416
Independent Reflections	1175 [R_{int} = 0.1266, R_{sigma} = 0.0445]	1164 [R_{int} = 0.1429, R_{sigma} = 0.0241]
R_{pim}	0.032	0.021
CC ½	0.992	0.993
Data/Restraints/Parameters	1175/0/36	1164/0/36
Goodness-of-fit on F^2	0.941	1.056
Final R indexes [I >= 2σ (I)]	R_1 = 0.0345, wR_2 = 0.0886	R_1 = 0.0438, wR_2 = 0.1208
Final R indexes [all data]	R_1 = 0.0399, wR_2 = 0.0903	R_1 = 0.0468, wR_2 = 0.1249
Largest diff. peak/hole e Å^{-3}	0.44/-0.33	0.38/-0.37

Table S11 Statistics of 22 crystals of **3** identified by photometric selection method (PS)

Metric	Mean	Standard Deviation	Mode	Median
I/σ(I)	7.1	3.3	4.3	6.1
R_{pim}	0.085	0.029	0.072	0.082
$CC_{1/2}$	0.977	0.072	0.997	0.994
Volume / $Å^3$	18156	118	18094	18163
α / °	90	0	90	90
β / °	90	0	90	90
γ / °	90	0	90	90
a / Å	26.28	0.057	26.25	26.29
b / Å	26.28	0.057	26.25	26.29
c / Å	26.28	0.057	26.25	26.29

Table S12 Statistics of 63 crystals of **3** identified by diffractive sweep method (DS)

Metric	Mean	Standard Deviation	Mode	Median
I/σ(I)	7.6	3.9	6.6	6.7
R_{pim}	0.092	0.034	0.086	0.086
$CC_{1/2}$	0.984	0.017	0.996	0.990
Volume / $Å^3$	18076	127	18197	18097
α / °	90	0	90	90
β / °	90	0	90	90
γ / °	90	0	90	90
a / Å	26.24	0.062	26.25	26.30
b / Å	26.24	0.062	26.25	26.30
c / Å	26.24	0.062	26.25	26.30

Table S13 Crystal size statistics as calculated from photometric data.

Sample	**1**	**2**	**3**
Mean cross sectional area of crystals / µm²	71.1	121	403
Mean crystal dimension / µm	6.8	7.2	18.4
Standard deviation of cross section area of crystals / µm²	87.0	237	358
Standard Deviation of crystal dimension / µm	5.0	8.4	8.3
Median cross sectional area of crystals / µm²	33.8	11.1	268
Median of crystal dimension / µm	5.8	3.3	16.3

Table S14 Breakdown of time taken for full data collection of **1** by PS and DS methods

**Photometric Selection (PS)**		**Diffracting Scan (DS)**	
Microscopy Collection Time /s	60	Number wells in grid scan	1296
Image Processing Time /s	120	Grid scan Collection Time per well (total) /s	0.5 (648)
		Overheads during collection per well (total) /s	3 (3888)
		Data processing time / s	120
Total Preprocessing Time / minutes	3	Total Preprocessing Time / minutes	63
Number 5° datasets	537	Number 5° datasets	292
Collection time per crystal (total) / s	5 (2685)	Collection time per crystal (total) / s	5 (1460)
Overheads during collection per well (total) / s	8 (4296)	Overheads during collection per well (total) / s	8 (2336)
Total Collection Time / minutes	116	Total Collection Time / minutes	128

Table S15 Breakdown for time take for full data collection of **2** by PS and DS methods

**Photometric Selection (PS)**		**Diffracting Scan (DS)**	
Microscopy Collection Time /s	60	Number wells in grid scan	1296
Image Processing Time /s	120	Grid scan Collection Time per well (total) /s	0.5 (648)
		Overheads during collection per well (total) /s	3 (3888)
		Data processing time / s	120
Total Preprocessing Time / minutes	3	Total Preprocessing Time / minutes	63
Number 5° datasets	296	Number 5° datasets	443
Collection time per crystal (total) / s	5 (1480)	Collection time per crystal (total) / s	5 (2215)
Overheads during collection per well (total) /s	8 (2368)	Overheads during collection per well (total) / s	8 (3544)
Total Collection Time / minutes	67	Total Collection Time / minutes	157

Table S16 Breakdown for time take for full data collection of **3** by PS and DS methods

**Photometric Selection (PS)**		**Diffracting Scan (DS)**	
Microscopy Collection Time	60	Number wells in gridscan	1296
Image Processing Time	120	Gridscan Collection Time per well (total) /s	0.5 (648)
		Overheads during collection per well (total) /s	3 (3888)
		Data processing time / s	120
Total Preprocessing Time / minutes	3	Total Preprocessing Time / minutes	63
Number 5° datasets	41	Number 5° datasets	66
Collection time per crystal (total) / s	5 (205)	Collection time per crystal (total) / s	5 (330)
Overheads during collection per well (total) /s	8 (328)	Overheads during collection per well (total) / s	8 (528)
Total Collection Time / minutes	12	Total Collection Time / minutes	75

Table S17 Comparing estimated experiment durations for PS and DS based on overheads and data collection parameters from Table S14-S16. Primary difference is the relative 'time cost' of the 36x36 grid scan required to cover the substrate when using a 100 micron beam size.

Number 5° Datasets	Duration of PS data collection / s	Duration of DS data collection / s	Duration DS / Duration PS
50	650	4430	6.8
100	1300	5080	3.9
150	1950	5730	2.9
200	2600	6380	2.5
250	3250	7030	2.2
300	3900	7680	2.0
350	4550	8330	1.8
400	5200	8980	1.7
450	5850	9630	1.7
500	6500	10280	1.6
550	7150	10930	1.5

Table S18 Comparing estimated experiment durations for PS and DS based on overheads and data collection parameters from Table S13-S15. Primary difference is the relative 'time cost' of the 96x96 grid scan required to cover the substrate when using a 40 micron beam size.

Number 5° Datasets	Duration of PS data collection / s	Duration of DS data collection / s	Duration DS / Duration PS
50	650	32906	50.6
100	1300	33556	25.8
150	1950	34206	17.5
200	2600	34856	13.4
250	3250	35506	10.9
300	3900	36156	9.2
350	4550	36806	8.1
400	5200	37456	7.2
450	5850	38106	6.5
500	6500	38756	6.0
550	7150	39406	5.5
